# Circular and unified analysis in network neuroscience

**DOI:** 10.7554/eLife.79559

**Published:** 2023-11-28

**Authors:** Mika Rubinov

**Affiliations:** 1 Departments of Biomedical Engineering, Computer Science, and Psychology, Vanderbilt University Nashville United States; 2 Janelia Research Campus, Howard Hughes Medical Institute Ashburn United States; Carnegie Mellon University United States; National Institute of Mental Health United States

**Keywords:** network neuroscience, systems neuroscience, computational neuroscience, statistical models, explanatory models, benchmark models, None

## Abstract

Genuinely new discovery transcends existing knowledge. Despite this, many analyses in systems neuroscience neglect to test new speculative hypotheses against benchmark empirical facts. Some of these analyses inadvertently use circular reasoning to present existing knowledge as new discovery. Here, I discuss that this problem can confound key results and estimate that it has affected more than three thousand studies in network neuroscience over the last decade. I suggest that future studies can reduce this problem by limiting the use of speculative evidence, integrating existing knowledge into benchmark models, and rigorously testing proposed discoveries against these models. I conclude with a summary of practical challenges and recommendations.

*You do not know anything until you know why you know it.*

Clovis Andersen, *The Principles of Private Detection* (McCall Smith, 2007), cited in Sokal, 2010.

## Introduction

Scientific models are explanations of reality (Shmueli, 2010; Frigg and Hartmann, 2020). Models come in many forms, from sentences to equations, and in many kinds, from hypotheses to theories. All models are false, but some models are truer than others (Mizrahi, 2020). Specifically, all else being equal, models that are more explanatorily successful — that explain the data more accurately or with fewer assumptions — are likely to be truer than rival models (Appendix 1).

Efforts to find truer models drive scientific progress but command relatively little neuroscientific attention. Neuroscience devotes greater efforts to produce better data or more replicable analyses (Frégnac, 2017). A study by Jonas and Kording, 2017 implicitly critiqued this imbalance of effort. The study showed that popular neuroscientific analyses of ideal data cannot explain the workings of a computer chip, a toy model of the nervous system. The study implied, in this way, that neuroscience must devote greater efforts to find truer models.

Science finds truer and truer models relative to stronger and stronger rival models. By contrast, many analyses in neuroscience test new speculative models against weak null models. Some of these analyses use circular reasoning to redundantly explain existing knowledge. These circular analyses of knowledge violate the principle of parsimony and, in this way, accept models that are less true relative to the strongest rival models. Here, I discuss the nature and prevalence of this problem in systems and network neuroscience. I show that the problem can confound key results and estimate that it is common in the network-neuroscience literature.

I suggest that studies can reduce this problem in three main ways. First, they can limit the use of speculative evidence. Second, they can integrate all important existing knowledge into benchmark models. Third, they can rigorously test the significance of proposed discoveries against these models. Together, these steps can reduce circular analyses, formalize existing knowledge, and benchmark future progress.

Much of the following discussion stresses the importance of unambiguous definitions. Accordingly, Table 1 defines the use of several potentially ambiguous technical terms.

**Table 1.** Definitions of terms.

Term	Definition
Principle of parsimony (Occam’s razor)	An assertion that all else being equal, models with fewer redundant features are likely to be truer than rival models (Baker, 2022). This assertion reflects an objective preference for parsimony rather than a subjective preference for simplicity or elegance. In this way, and contrary to misconception, the principle of parsimony does not imply that reality, or its truest models, are simple or elegant.
Trueness (bias)	Distance between expected and true estimates of model parameters (ISO, 1994). True values of model parameters are typically inaccessible, and trueness (bias) can therefore be defined only in relative terms. The principle of parsimony asserts that all else being equal, models with fewer redundant features have truer (less biased) parameter estimates relative to rival models.
Precision (variance)	Expected distance between repeated estimates of model parameters (ISO, 1994). Precision (variance) does not require knowledge of the true values of model parameters and can therefore be defined in absolute terms. The problem of irreplicable results (Ioannidis, 2005) is primarily a problem of precision (variance).
Circular analysis	An analysis that first tests a model in a way that almost invariably accepts the model and then accepts the model on the basis of this test. This definition includes circular analyses of knowledge that accept overspecified models or redundant (less true) explanations. It also includes circular analyses of noise that accept overfitted models or irreplicable (less precise) explanations (Kriegeskorte et al., 2009).
Neural circuits or brain networks	Groups of connected neurons or brain regions that mediate function. This definition does not intend to make analogies between groups of neurons or brain regions, and electronic circuits or artificial neural networks (Rubinov, 2015).
Function	Behavior and other action that helps animals to survive and reproduce (Roux, 2014). This definition excludes physiological phenomena that lack such useful action.
Structure	Anatomical or physiological organization. This definition encompasses all physiological phenomena, including phenomena that lack known function.
Development	Formation of structure before and after birth. This definition includes plasticity and therefore encompasses learning and memory.

### General definitions

Analyses of complex datasets are vulnerable to distortions by extraneous features. Such distortions may include corruption by noise or confounding by existing knowledge. Statistical science, machine learning, and other fields have developed rigorous tests to mitigate the risk of these distortions. Analyses of complex datasets that neglect such tests, however, will almost invariably be distorted by extraneous features to some extent.

These distortions can generally lead to inflated agreement between model and data and to inappropriate model acceptance on the basis of this inflated agreement. The nature of individual distortions, however, will ultimately determine the individual consequences of this problem. On the one hand, corruption of analyses by noise can lead to the well-known problem of model overfitting and to irreplicable explanations (Kriegeskorte et al., 2009; Vul et al., 2009). On the other hand, confounding of analyses by existing knowledge can lead to a distinct, and less well-known, problem of model overspecification and to redundant explanations.

This work describes analyses that neglect to test speculative models against existing knowledge and that consequently accept overspecified models and redundant explanations. This section first defines the nature of this problem and then outlines a general solution.

#### Toy analogy

**Figure 1.**
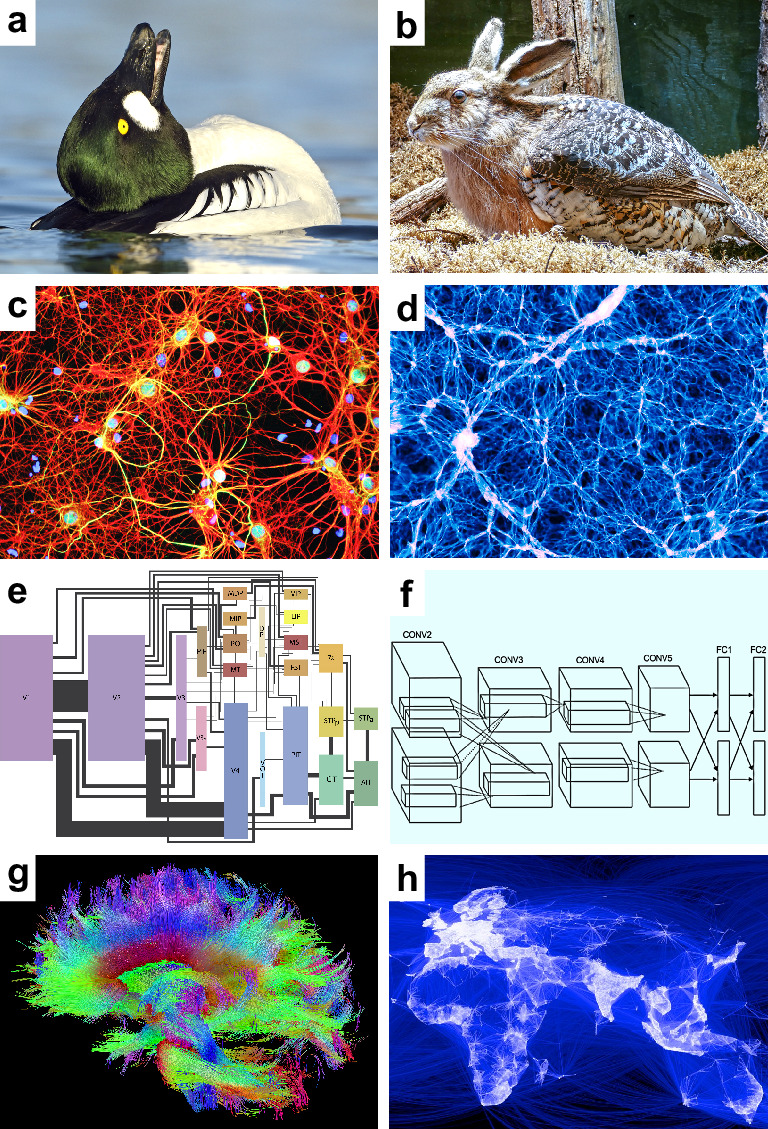
Speculative models. Speculative hypotheses that rest on apparent similarities between (**a**) an ambiguous duck-rabbit animal and (**b**) a skvader, a type of winged hare; (**c**) networks of neurons and (**d**) networks of galaxies; (**e**) a cortical visual system and (**f**) a convolutional neural network, a machine learning model for classifying images; (**g**) large-scale brain networks and (**h**) global friendship networks. Panel (a) is reproduced from Tim Zurowski (Shutterstock). Panel (b) is reproduced from Gösta Knochenhauer. Panel (c) is reproduced from Figure 4.2 of Stangor and Walinga, 2014. Panel (d) is adapted from the Illustris Collaboration (Vogelsberger et al., 2014). Panel (e) is reproduced from Figure 1 of Wallisch and Movshon, 2008. Panel (f) is adapted from Figure 2 of Krizhevsky et al., 2012. Panel (g) is reproduced from the USC Laboratory of NeuroImaging and Athinoula A. Martinos Center for Biomedical Imaging Human Connectome Project Consortium. Panel (h) is reproduced from Paul Butler (Facebook).

We can get an intuition for the problem with a toy analysis of a biological image (Figure 1a). The image is ambiguous, but our existing biological knowledge tells us that it most likely shows a duck — specifically a male duck doing a head-throw, its signature courting move. Sometimes our analyses may neglect such knowledge. This neglect will not make knowledge disappear. Instead, it will inflate the importance of hypotheses redundant with this knowledge.

We may propose, for example, that the image shows a skvader, a type of winged hare (Figure 1b). Our existing knowledge makes this hypothesis redundant — ducks doing head-throws almost always look like skvaders. Our neglect of this knowledge, however, can make the hypothesis seem important. We may accept the hypothesis on the basis of this perceived importance. This acceptance, however, will lead to redundant explanations. We will implicitly “double dip” or explain the same image twice — first as a duck and second as a skvader.

#### Circular analysis

We can define the problem more formally with three types of models.

*Benchmark models (well-specified models)*. These models represent all important existing knowledge about our phenomenon of interest. They include all benchmark features, features of known importance to this phenomenon, and they exclude all other features. In systems neuroscience, benchmark features often represent existing knowledge about the function, structure, development, and evolution of neural circuits. Distinct phenomena may have distinct benchmark models, and one phenomenon may have several competing benchmark models.

*Speculative models*. These models represent new hypotheses about some phenomenon of interest. They include one or more speculative features, features of possible but uncertain importance to this phenomenon. Some speculative features may turn out to be redundant with benchmark features. For example, consider the similarity of the human brain and the universe (Figure 1c–d). Both systems have billions of nested, spatially embedded, and interacting elements: neurons and galaxies (Vazza and Feletti, 2020). Let the feature of *cosmicity* denote the resemblance of a complex system to the universe. The human brain has high cosmicity. A speculative model may propose, on this basis, that brain dynamics resemble cosmic dynamics. Note, however, that brain cosmicity is likely to be redundant with our existing knowledge about the structure of neural circuits.

*Strawman models (underspecified models).* These models represent weak null hypotheses. They typically exclude the benchmark features with which the speculative features are redundant. In our example, a strawman model excludes the known structure of neural circuits with which cosmicity is redundant.

*Circular analyses.* These analyses almost invariably accept speculative models against strawman models (Box 1, Appendix 2). They comprise circular analyses of noise and circular analyses of knowledge (Appendix 3). Circular analyses of noise, the focus of previous work (Kriegeskorte et al., 2009), result in acceptance of noisy or irreplicable explanations. By contrast, circular analyses of knowledge, the focus of this work, result in acceptance of redundant explanations. In our example, a circular analysis of knowledge will almost invariably accept the significance of cosmicity against our strawman model. The analysis will be circular because our strawman model excludes the known structure of neural circuits with which cosmicity is redundant.

Box 1. **A classification of circular analyses.**

**General definition (weak evidence of progress)**

Circular analyses are analyses that use circular reasoning. These analyses:

1. Test a model in a way that almost invariably accepts the model.
2. Accept the model on the basis of this test.

In general, circular analyses denote weak evidence of progress but do not necessarily preclude progress. In this way, these analyses do not necessarily denote strong evidence of stagnation. These analyses also violate Mayo’s weak-severity requirement of “bad evidence, no test” (Mayo and Spanos, 2011; Mayo, 2018; Appendix 2).

**Specific definition (strong evidence of stagnation)**

This work describes circular analyses of knowledge. These analyses:

1. Test a speculative model in a way that almost invariably accepts it against a strawman model. Specifically, these analyses test the statistical significance of speculative features in a way that almost invariably shows the significance of these features against a strawman model because:
  - The speculative features are redundant with one or more benchmark features.
  - The strawman model excludes the benchmark features with which the speculative features are redundant.
2. Accept the speculative model on the basis of this test.

Circular analyses of knowledge explain the same aspect of the data twice: first, as one or more benchmark features and second, as a speculative feature redundant with these benchmark features. In this way, these analyses necessarily denote strong evidence of stagnation. Note that in principle, the acceptance of redundant explanations may signify regress rather than mere stagnation. In practice, however, the relatively transient nature of many such explanations suggests that stagnation is a more apt description of the problem, cf. “[w]hen we examine the history of favored stories for any particular adaptation, we do not trace a tale of increasing truth as one story replaces the last, but rather a chronicle of shifting fads and fashions.” (Gould, 1978)

*Analyses of noise and analyses of knowledge*. Previous work has described circular analyses of noise (Kriegeskorte et al., 2009). These analyses have deep similarities with circular analyses of knowledge. Both analyses center on the problem of false discovery and are equivalent in other important respects (Appendix 3).

*Redundant explanations (overspecified models).* Studies sometimes conclude that speculative features should replace or overturn the benchmark features with which they are redundant. Circular analyses of knowledge cannot support such conclusions because they never test the speculative features against a benchmark model. Such analyses must therefore accept, often implicitly, a model that includes all the existing benchmark features and the redundant speculative features.

In our example, we do not test cosmicity against existing knowledge with which it is redundant and so cannot overturn this existing knowledge. Our analysis implies, therefore, that cosmicity enriches, but does not replace, our existing knowledge. In this way, we must accept the importance of cosmicity and simultaneously accept the importance of existing knowledge with which cosmicity is redundant.

This problem extends to the acceptance of many, potentially countless, speculative models against the same strawman model. Such acceptance implicitly proposes the simultaneous importance of many, potentially countless, redundant features. Moreover, the circular acceptance of one speculative model after another can give an impression of progress even as it leads to stagnation.

#### Unified analysis

**Figure 2.**
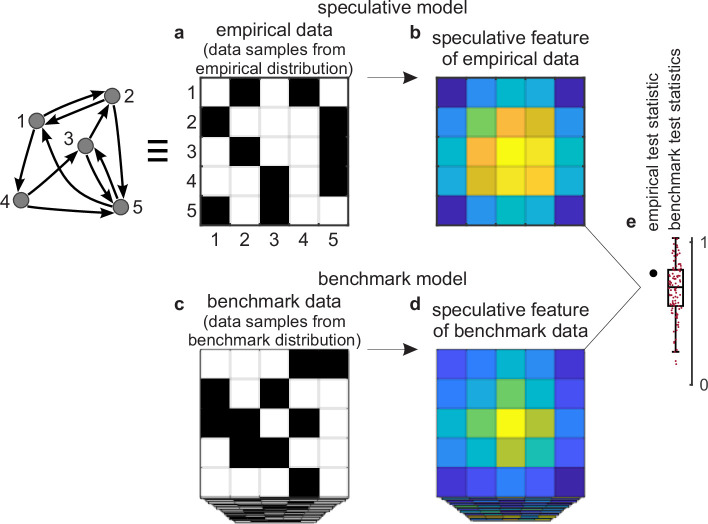
Tests against benchmark models. (**a**) An empirical data sample. The diagram (left) shows a network representation of this sample. This example shows only one empirical data sample, but in general there could be many such samples. (**b**) A speculative feature computed on empirical data. In this example, the feature has the same size as the data, but in general it could have an arbitrary size. Colors denote values of feature elements. (**c**–**d**) Corresponding (**c**) benchmark data samples and (**d**) speculative features computed on these data. (**e**) Empirical test statistic (large black dot) and corresponding benchmark test statistics (small red dots). The effect size reflects the deviation of the empirical test statistic from the benchmark test statistic. The uncertainty (confidence) interval and p-value reflect the statistical significance of this deviation. This panel shows a non-significant effect and thus implies that the speculative feature does not transcend the benchmark model of existing knowledge.

A general solution to this problem centers on significance tests of speculative features against benchmark models (Figure 2). These tests represent unified analyses of existing knowledge and proposed discovery. They form controlled experiments that test the importance of one feature by controlling for the effects of all known confounding features (Sibbald and Roland, 1998; Box 2). They also form a type of *severe (model) selection* within Mayo’s framework of *severe testing (*Mayo and Spanos, 2011; Mayo, 2018; Appendix 2). Finally, they parallel controls for model overfitting (Kriegeskorte et al., 2009; Appendix 3).

Box 2. **Tests against benchmark models and randomized controlled trials.**

Tests against benchmark models have deep similarities with randomized controlled trials, controlled experiments in medical research (Sibbald and Roland, 1998). Randomized controlled trials comprise three main steps. The first step randomly splits a sample of people into a treated group and a control group. The second step gives the treatment to people in the treated group and gives a placebo to people in the control group. The third step compares the medical outcomes of the two groups.

The following list shows that tests against benchmark models (or *tests*) have essentially the same structure as randomized controlled trials (or *trials*), even as they differ in implementational details.

- Samples of empirical data (in *tests*) parallel people in the treated group (in *trials*).
- Samples of benchmark-model data (in *tests*) parallel people in the control group (in *trials*).
- Comparison of test statistics (in *tests*) parallels comparison of medical outcomes (in *trials*).
- Maximally random, or unbiased, sampling of benchmark-model data (in *tests*) parallels maximally random, or unbiased, split into the treated and control groups (in *trials*). Both approaches allow, in principle, to control for all known (*tests*) or all possible (*trials*) confounding explanations.

Despite these similarities, these two types of experiments have one basic difference. Randomized controlled trials can test causality because the treatment always precedes the outcome in time (Siddiqi et al., 2022). By contrast, tests against benchmark models can test non-redundancy but do not test causality unless we have additional information about the temporal precedence of speculative and benchmark features.

In practice, these analyses center on the sampling of data from benchmark-model distributions and on the testing of speculative features against these data. We can describe these analyses in three steps.

First, we can consider a sample of empirical data. The sample could be as small as a single dataset (Figure 2a) or it could be larger. We can compute a speculative feature of interest on this sample and summarize this feature with a test statistic (Figure 2b and e). The empirical test statistic reflects the importance of the corresponding speculative feature. It can also reflect, by extension, the importance of the speculative model that centers on this feature.

Second, we can get many data samples from a benchmark-model distribution (Figure 2c). These samples should match the statistics of all benchmark features but be maximally random in all other respects. We can compute the test statistic on these samples and in this way estimate the null distribution — the distribution of the test statistic under the null hypothesis of existing knowledge (Figure 2d and e).

Third, we can test the significance of the empirical test statistic against this null distribution by estimating the effect size, uncertainty (confidence) interval, and p-value (Mayo and Spanos, 2011). The p-value can reflect the probability that the empirical test statistic does not exceed the benchmark test statistic. In this way, and with appropriate definitions of the test statistic and the benchmark model, the p-value can reflect the probability that our proposed discovery does not transcend existing knowledge.

In our cosmicity example, we can do this analysis in three steps. First, we can define a test statistic of cosmicity and estimate the empirical value of this statistic. Second, we can define a benchmark model that includes all important existing knowledge about the structure of neural circuits. We can then sample data from this model distribution and estimate the null distribution of the test statistic. Third, we can use this null distribution to estimate the effect size, the uncertainty interval, and the p-value, and in this way test the significance of cosmicity against our existing knowledge of neural circuits.

As we discussed above, cosmicity is likely to be redundant with our existing knowledge. This likely redundancy suggests that our result is unlikely to be statistically significant. In this context, a finding of statistical significance can serve as genuine evidence for the importance of cosmicity and, by extension, for the importance of cosmic dynamics to brain function.

### Specific examples

Previous work has noted that circular analyses of noise can be “hard to understand, imagine, or predict” and “when it’s hard to see how, it can still be happening” (Kriegeskorte et al., 2009). This section shows that circular analyses of knowledge can often be similarly inconspicuous. It first describes possible examples of these analyses in systems neuroscience and probable examples in network neuroscience. It then walks through the details of the problem with a toy analysis. It finally estimates the prevalence of the problem in the network-neuroscience literature.

#### Possible circular analyses of knowledge

Systems neuroscience broadly studies the structure and function of interacting groups of neurons or brain regions. The field variously terms these groups assemblies, populations, circuits, systems, or networks. It has acquired considerable, albeit somewhat scattered, knowledge about the structure and function of these groups. It has also proposed many speculative hypotheses that seek to transcend this existing knowledge.

We can show how circular analyses of knowledge can lurk in this environment using the example of the systems neuroscience of (mammalian) vision. In line with our discussion, we can first consider the benchmark, speculative, and strawman models of this phenomenon.

*Benchmark model.* Systems neuroscience lacks a benchmark model that captures our existing knowledge about the nature and origin of vision (Poggio and Serre, 2013; Golan et al., 2023). Despite this lack of a benchmark model, we know many benchmark features relevant to vision. We know, for example, that the visual system tightly balances the activity of inhibitory and excitatory neurons (Isaacson and Scanziani, 2011). This balance prevents overinhibition and overexcitation and thus allows animals to sense light and not get seizures (Ma et al., 2019). We also know that this balance rests, in part, on the fast-spiking response of inhibitory neurons to excitatory visual stimulation (Sohal, 2016). Finally, we know that vision evolved, in virtually all animals, to support visuo-motor interactions, that is, to help animals interact with their environments through movement (Goodale, 1996; Nilsson, 2021). These basic features do not necessarily form a benchmark model, but they will suffice for our discussion.

*Speculative models.* Systems neuroscience has many speculative models of vision. Many of these models center on the importance of elegant features and often rest on analogies with other natural and synthetic systems. We can consider three prominent examples of these models.

The first model centers on the importance of *internal representations*, patterns of neuronal activity that internally represent visual stimuli (Craik, 1943; Hubel and Wiesel, 1959). Studies have proposed that the visual system interprets the meaning of internal representations much like an artificial neural network decodes the nature of input images (Kriegeskorte, 2015; Richards et al., 2019; Cichy and Kaiser, 2019; Figure 1e–f). Despite these intuitions, we have no evidence that patterns of neuronal activity actually denote internal representations (Kenny, 1971; Brette, 2019; Bennett and Hacker, 2022). Moreover, in many cases, we may be able to explain these patterns as substrates of visuo-motor interactions without the need to assume that they internally represent anything at all (Freeman and Skarda, 1990; Cao, 2020; Driscoll et al., 2022).

The second model centers on the importance of *gamma oscillations*, fast rhythms of neuronal activity that correlate with visual perception (Gray et al., 1989; Burwick, 2014). Studies have proposed that gamma oscillations bind simple visual stimuli into complex perception, much like orchestra conductors weave the sounds of individual musicians into complex music (Singer, 2001; Buzsáki and Draguhn, 2004). Despite these intuitions, we know that gamma oscillations are absent during the perception of some images, and so may not be necessary to bind stimuli into perception (Hermes et al., 2015b; Hermes et al., 2015a). Moreover, in many cases, we may be able to explain these oscillations as the inevitable outcomes of inhibitory responses to visual stimulation without the need to assume that they bind anything at all (Ray and Maunsell, 2015; Singer, 2018).

The third model centers on the importance of neural criticality, collective neuronal activity that balances on the edge of order and disorder. Studies have proposed that criticality optimizes our sensitivity to visual stimuli, much like the critical (neither shallow nor steep) angle of a sand pile optimizes its responsiveness to tactile stimuli (Shew et al., 2009; Shew and Plenz, 2013). Despite these intuitions, we know that signatures of criticality can occur in the absence of any visual stimuli and so may not necessarily be related to optimized visual sensation (Fontenele et al., 2019; Destexhe and Touboul, 2021). Moreover, in many cases, we may be able to explain these signatures as inevitable outcomes of balanced inhibitory and excitatory activity without the need to assume that they optimize anything at all (Nanda et al., 2023).

*Strawman models.* We cannot summarize the full range of null models in the expansive literature of representations, oscillations, and criticality. We can still do justice to this literature, however, by considering some of its strongest models. One such model can test the significance of representations against correlations of neuronal activity across space and time (Elsayed and Cunningham, 2017). Another model can test the significance of oscillations against non-oscillatory activity of similar amplitude (Donoghue et al., 2022). A third model can test the significance of critical neuronal activity against mimicking non-critical (lognormal) phenomena (Buzsáki and Mizuseki, 2014). Together, all these models can test representations, oscillations, and criticality against important confounders. Despite this, none of these models test these speculative features against the benchmark features with which they may be redundant.

*Circular analyses and redundant explanations.* Tests against strawman models often accept the importance of representations, oscillations, and criticality. Separately, these tests cannot reject the importance of benchmark features with which these speculative features may be redundant. It follows that these tests may implicitly explain the same aspects of brain activity twice — first as a basic benchmark feature and second as a redundant speculative feature. In the study of vision, these analyses may therefore conclude the simultaneous importance of:

1. Visuo-motor interactions and internal representations possibly redundant with these interactions.
2. Inhibitory responses to stimulation and gamma oscillations possibly redundant with these responses.
3. Balance of inhibition and excitation and critical activity possibly redundant with this balance.

Individually, these analyses accept simple or elegant models. Collectively, however, they may accept a needlessly complicated model that assumes the simultaneous importance of several redundant features.

#### Probable circular analyses of knowledge

Many parts of systems neuroscience, such as the study of vision, lack well-defined benchmark models or the ability to test speculative models against these benchmarks. These limitations make it hard to show the presence of circular analyses of knowledge, even when they exist.

Some parts of systems neuroscience, however, have relatively well-defined benchmark models and the ability to test speculative models against these benchmarks. These strengths make it possible to show the presence of circular analyses of knowledge when they exist. Here, we can describe the probable presence of such analyses in network neuroscience.

Network neuroscience is a subfield of systems neuroscience that studies the structure and function of extensive, including whole-brain, networks (Bassett and Sporns, 2017). Nodes in these networks typically denote cells or regions, while links typically denote synapses, axonal projections, or activity correlations. We can show probable circular analyses in this field using the example of the network neuroscience of (mammalian) cortex. In line with our previous discussion, we can first consider the benchmark, speculative, and strawman models of this structure.

**Figure 3.**
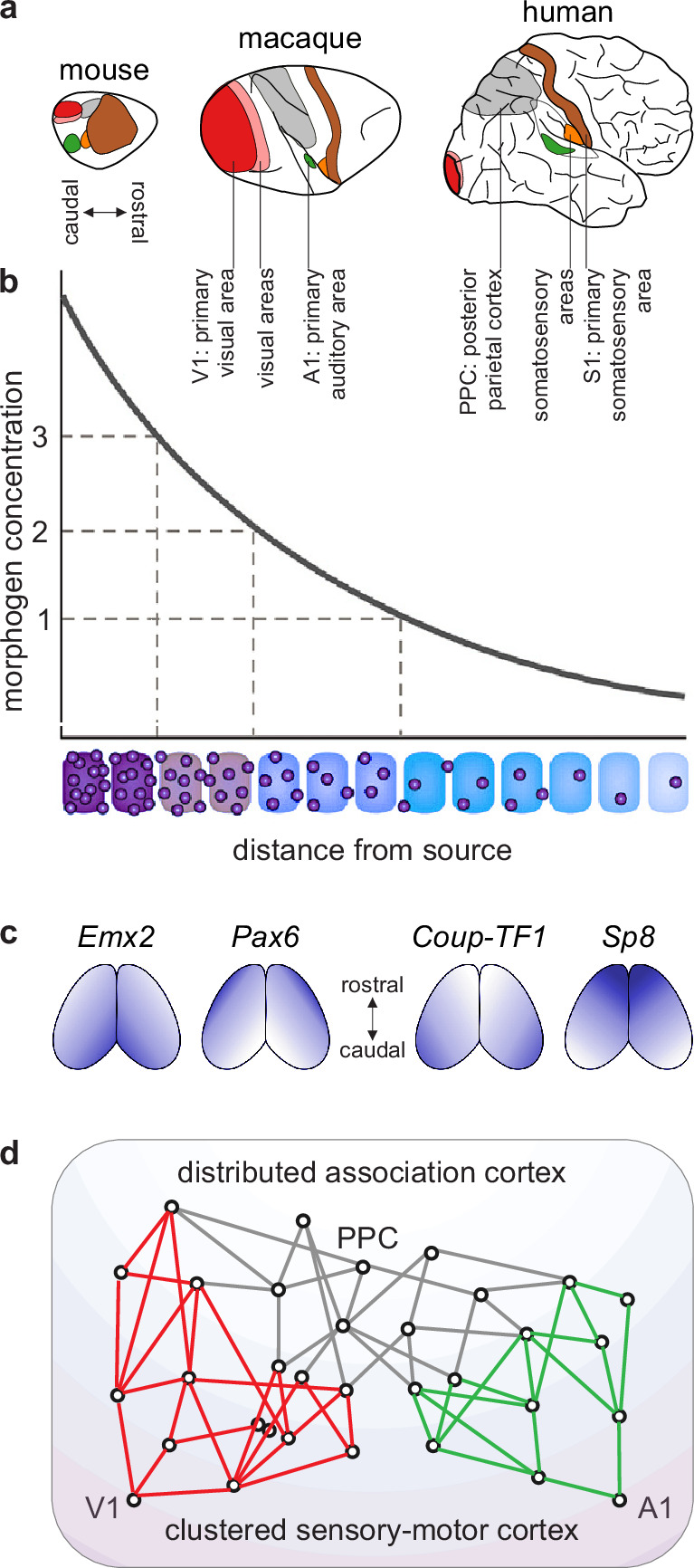
A blueprint of large-scale cortical networks. (**a**) Rostrocaudal (nose-to-tail) maps of shared cortical regions in three popular mammalian model organisms. Virtually all mammals have well-delineated primary and other sensory areas, and an ill-delineated posterior parietal association cortex. In addition, most mammals have well-delineated primary and other motor areas (not highlighted in this panel). (**b**–**c**) Gradients of cortical development. (**b**) Spatial gradients of morphogen concentration induce corresponding (**c**) spatial gradients of transcription-factor and gene expression. Morphogens are signaling molecules that establish spatial concentration gradients through extracellular diffusion from specific sites. Transcription factors (names in italics) are intracellular proteins that establish spatial gradients of gene expression. The discretization of these gradients during development results in the formation of discrete cortical areas and systems (colors in b). (**d**) A schematized blueprint of a macaque cortical network reflects a gradual transition of a relatively clustered sensory-motor cortex (red and green) into a relatively distributed association cortex (gray). Circles denote cortical regions, while lines denote interregional projections. V1 and A1 denote primary visual and auditory areas, while PPC denotes posterior parietal association cortex. Panel (a) is adapted from Figure 3 of Krubitzer and Prescott, 2018. Panel (b) is adapted from Figure 1.3b of Grove and Monuki, 2020. Panel (c) is adapted from Figure 2 of Borello and Pierani, 2010. Panel (d) is adapted from Figure 2d of Mesulam, 1998.

*Benchmark model.* We have considerable knowledge of evolution, development, structure, and function of cortical networks. First, *evolutionary* analyses of extensive mapping studies suggest that essentially all mammals share a common cortical blueprint (Kaas, 1995; Krubitzer, 1995; Figure 3a). Second, the commonality of this blueprint likely stems from strongly conserved *developmental* processes. These processes include an initial establishment of spatial concentration gradients of developmental molecules and a subsequent discretization of these gradients (Figure 3b–c). Third, signatures of these developmental processes show through in the *structure* of the adult cortex. To a first approximation, this structure reflects a gradual transition along the cortical sheet (Figure 3d):

- from a relatively well-delineated, clustered, and poorly connected sensory-motor cortex.
- to a relatively ill-delineated, distributed, and highly connected association cortex.

(The sensory-motor cortex is well-delineated in large part because it comprises cortical areas that form spatial mappings of entire sensory or motor fields. For example, the primary somatosensory area comprises a spatial mapping of all body parts that can receive somatic input. By contrast, the association cortex is ill-delineated in large part because it lacks areas that form similarly clear mappings of complete sensory or motor fields [Buckner and Krienen, 2013; Patel et al., 2014].)

Fourth, this cortical structure constrains known cortical *function*. Specifically, a gradual transition from a relatively well-delineated sensory-motor cortex to a relatively ill-delineated association cortex reflects a corresponding transition from relatively well-defined sensory-motor function to relatively ambiguous cognitive function (Bayne et al., 2019).

Network neuroscience has a well-known model that captures the basic features of this cortical blueprint (Sporns, 2013). This model includes two types of benchmark features. First, it includes network modules (clusters) that capture the clustered sensory-motor cortex. Second, it includes node connectivity (number of connections) that captures the gradual transition from the poorly connected sensory-motor cortex to the well-connected association cortex. We can adopt this basic benchmark model for our subsequent discussion.

*Speculative models.* Speculative models in network neuroscience broadly resemble other speculative models in systems neuroscience. These models center on the importance of elegant features and often rest on analogies with other natural and synthetic systems, such as metabolic, transport, and friendship networks (Barabási, 2016; Figure 1g–h). In contrast to broader systems neuroscience, however, speculative features in network neuroscience are often more clearly redundant with benchmark features. We can show this redundancy in three speculative models that reflect some of the best-known results in network neuroscience.

The first model centers on the importance of small-world structure (Stephan et al., 2000; Achard et al., 2006). This structure denotes the simultaneous presence of many network triangles (triplets of fully connected nodes) and many network shortcuts (connections between different network parts). Studies have proposed that small-world cortical structure optimizes the competing demands of functional segregation and integration (Sporns and Zwi, 2004). We also know, however, that this structure is redundant with connected sensory-motor modules: “modular systems are small-world but not all small-world systems are modular” (Meunier et al., 2010).

The second model centers on the importance of cores or clubs (Hagmann et al., 2008; Zamora-López et al., 2010; van den Heuvel and Sporns, 2011). These structures denote groups of highly connected nodes. Studies have proposed that cores or clubs of the association cortex form the backbone of functional integration and may underpin the global workspace, a theoretical substrate of consciousness (Griffa and van den Heuvel, 2018). We also know, however, that these structures are redundant with sensory-motor modules and highly connected association nodes (hubs): “clubs are structural byproducts of modules and hubs” (Rubinov, 2016).

The third model centers on the importance of node controllability (Tang et al., 2012; Gu et al., 2015). High-control nodes in dynamical systems mediate switches between network activity (system states). Studies have proposed that high-control cortical nodes may support internal cognitive control and may serve as levers for external cortical control (Tang and Bassett, 2018). We also know, however, that node controllability is roughly equivalent with node connectivity (degree) (Tu et al., 2018) or related features (Patankar et al., 2020): “a strong […] correlation between node degree and average controllability is mathematically expected” (Gu et al., 2015).

*Strawman models.* Studies of small worlds, cores/clubs, and controllability use a relatively limited set of null models. First, tests of small worlds and cores/clubs tend to follow the broader network-science literature and use null models that include node connectivity but not network modules (Watts and Strogatz, 1998; Colizza et al., 2006). Second, many tests of controllability use abstract null models that lack node connectivity or network modules (Pasqualetti et al., 2019). Third, many studies also use null models that include the empirical decay of connectivity with spatial distance (Markov et al., 2013). These spatial models can account for much variance in the data and are perhaps the strongest network-neuroscience null models in common use today (Kaiser and Hilgetag, 2006). Despite these strengths, these models lack node connectivity or network modules and cannot compete with benchmark models that include these features (Rubinov, 2016).

*Circular analyses and redundant explanations.* Tests of small worlds, cores/clubs, and controllability against strawman models will almost invariably accept the importance of these speculative features. Separately, these tests cannot reject the importance of benchmark features with which these speculative features are redundant. It follows that these circular analyses implicitly explain the same aspects of network structure twice: first as a basic benchmark feature, and second as a redundant speculative feature. Individually, these analyses accept simple or elegant models. Collectively, however, they accept a needlessly complicated model that assumes the simultaneous importance of sensory-motor modules, highly connected association nodes, small worlds, cores/clubs, and controllability.

#### Walkthrough circular analysis of knowledge

**Figure 4.**
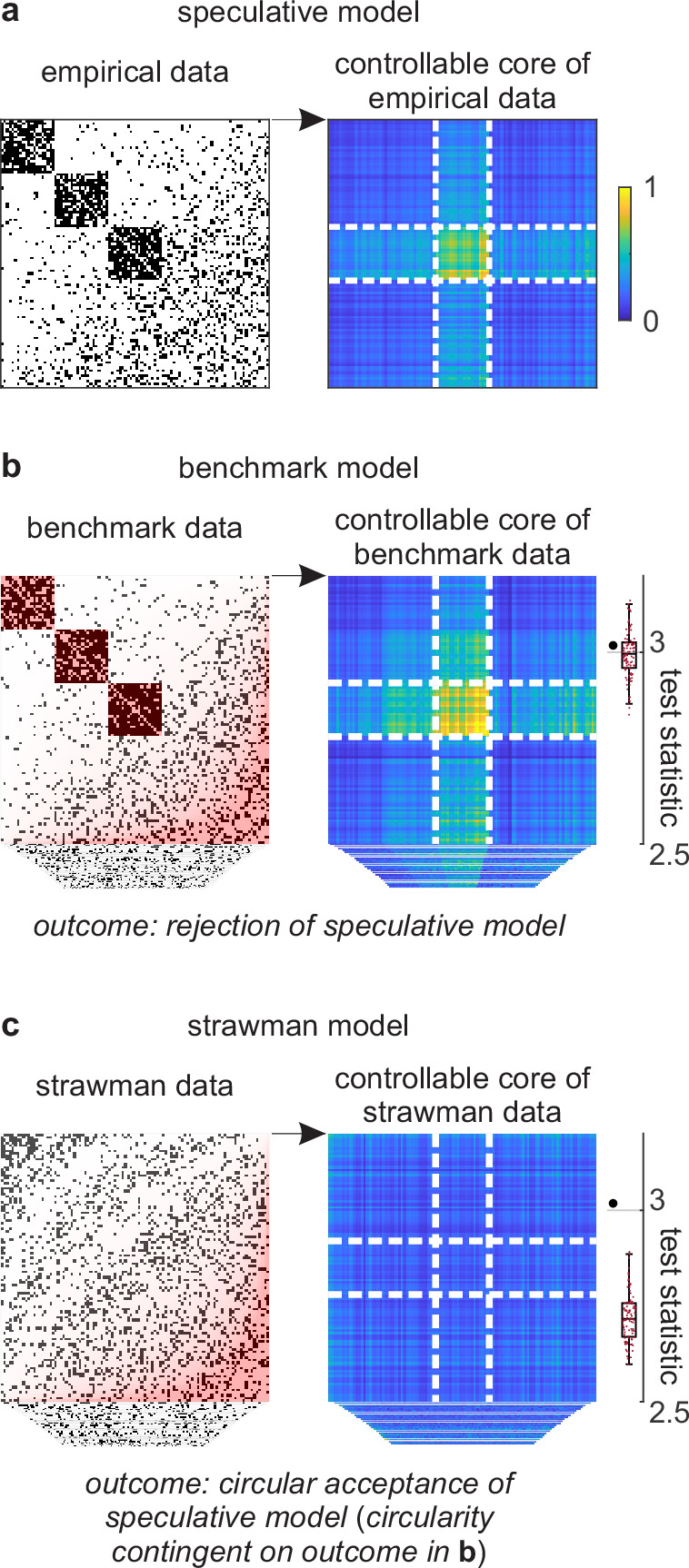
Example analysis. (**a**) Left: A toy cortical network. Right: A matrix that reflects the controllability of specific network states (a one-rank approximation of the controllability Gramian [Brunton and Kutz, 2019]). Dashed lines delineate the controllable core. The test statistic is the logarithm of the sum of all matrix elements within this core. (**b**) Left: Data samples from a benchmark-model distribution. The benchmark model includes empirical network modules and node connectivity (red overlays). Right: Controllable cores in benchmark-model data. Rightmost: Empirical (large black dot) and benchmark test statistics (small red dots). (**c**) Left: Data samples from a strawman model distribution. The strawman model includes node connectivity but not empirical network modules (red overlay). Right: Controllable cores in strawman-model data. Rightmost: Empirical test statistic (large black dot) and strawman test statistics (small red dots).

We can show the details of this problem with a walkthrough analysis of a toy cortical network. This network has an accentuated transition from clustered to distributed cortical connectivity (Figure 4a–b, left). We can propose a speculative model of this network that centers on a toy feature of a controllable core. This hybrid feature represents a core of cortical regions whose activity can be induced with relatively little stimulation. Theory suggests that this controllable core may support a stable state of cortical activity and thus play an important role in cortical function. Despite these considerations, the existence and importance of this feature remain speculative without tests against a benchmark model.

We can test this feature against a benchmark model in three steps. First, we can define a test statistic that reflects the importance of this feature. In our example, we can define this statistic to be the core density of controllable network nodes (Figure 4a, right). Second, we can compute the value of this statistic on empirical and benchmark-model data (Figure 4b). Third, we can use these values to quantify the effect size, uncertainty interval, and p-value. In our analysis, the empirical test statistic is 3.02, while the median [95% uncertainty interval] benchmark-test statistic is 3.00 [2.87, 3.12] (arbitrary units). The corresponding effect size of 0.02 [−0.11, 0.15] and p=0.36 (Figure 4b, right) suggest that the empirical test statistic is not significant against benchmark-model data. This analysis suggests that the controllable core is redundant with our existing knowledge.

Separately, we can test the significance of a controllable core against a strawman model. (Figure 4c). In our analysis, the strawman-model statistic is 2.72 [2.62, 2.88]. The corresponding effect size of 0.30 [0.14, 0.40] and p<0.01 (Figure 4c, right) suggest a rejection of this strawman model. This rejection is circular because the strawman model excludes the benchmark feature with which the controllable core is redundant.

#### Prevalence of probable circular analyses of knowledge

I quantified the fraction and number of probable circular analyses of knowledge in the network-neuroscience literature. I did this by evaluating network-neuroscience studies published during five recent years in ten journals. Appendix 4 describes the details of this evaluation.

This evaluation shows that 56% of evaluated studies had at least one circular analysis of knowledge. A simple extrapolation suggests that this problem may have affected more than three thousand original studies published over the last decade. This extrapolation is necessarily a rough estimate. It may be upwardly biased if my sample is unrepresentative of the broader literature or downwardly biased if my search criteria missed other affected articles. Despite these limitations, this extrapolation forms a useful indicator of the magnitude of this problem in the literature.

I did not try to assess the effects of this problem on individual results. These effects will depend on the aims and conclusions of individual studies. For example, circular analyses of knowledge in some studies may be tangential to the main results and may not affect the main conclusions. Separately, circular analyses in other studies may make the main results seem falsely novel or important and, in this way, may severely distort the main conclusions. Overall, I agree with a previous similar evaluation of the literature (Kriegeskorte et al., 2009) that such effects should be assessed through systematic community efforts.

To facilitate these efforts, I created a semi-automated analysis pipeline that downloads and curates all published studies that match some specified search criteria (Appendix 4). The curation includes the extraction of the Methods and Results sections and the highlighting of possible descriptions of benchmark, speculative, or strawman models. This basic curation cannot replace the careful evaluation of individual articles, but it may help to make such an evaluation standardized and more objective.

### Speculative evidence

The commonness of circular analyses of knowledge may reflect, in part, the intuitive importance of many speculative models. This importance often rests on the misleading suggestiveness of speculative evidence. The ability to spot such evidence can help to shift the focus from speculative intuitions to rigorous tests and, in this way, alleviate much of this problem in the literature.

This section discusses how suggestive terminology, suggestive structure, and suggestive narratives can all falsely signal the importance of speculative features. This discussion aligns with similar perspectives in neuroscience (Krakauer et al., 2017), machine learning (Lipton and Steinhardt, 2019), and psychology (Yarkoni, 2020).

#### Suggestive terminology: Deepities

The term *deepity* denotes a word or phrase that has two distinct meanings (Dennett, 2013). The first meaning is direct and undisputed but bland, while the second is profound but indirect and speculative. Deepities do damage when they lead us to conflate the two meanings and, in this way, make speculative or redundant features seem well-supported.

Many bedrock terms or ideas in systems neuroscience are deepities because they conflate facts with speculations (Table 2). Here, we can show this conflation using three especially consequential terms: *function*, *emergence*, and *significance*. We can do so using a toy example of “lub-dub” heart sounds, features that arise as byproducts of turbulent blood flow.

**Table 2.** Example deepities.

Deepity	Direct meaning	Implicit allusion
Neural computation (Churchland and Sejnowski, 2016)	Transformation of sensory input to behavioral output.	Computer-like transformation of sensory input to behavioral output.
Neural representation, code, or information (Baker et al., 2022; Brette, 2019; Nizami, 2019)	Patterns of neuronal activity that correlate with, or change in response to, sensory input.	Internal representations or encodings of information about the external world.
Neural networks (Bowers et al., 2022)	Artificial neural networks (machine-learning models).	Biological neural networks.
Necessity and sufficiency (Yoshihara and Yoshihara, 2018)	The induction or suppression of behavior through stimulation or inhibition of neural substrate.	Logical equivalence between behavior and neural substrate.
Functional connectivity (Reid et al., 2019)	Correlated neural activity.	Neural connectivity that causes function.
Complexity (Merker et al., 2022)	Patterns of neural structure that are neither ordered nor disordered.	Patterns of neural structure that are fundamentally important.
Motifs	Repeating patterns of brain-network connectivity.	Motifs of neural computation.
Efficiency	Communication between pairs of brain nodes via algorithmic sequences of connections.	Efficiency of neural communication.
Modularity	Propensity of brain networks to be divided into clusters.	Propensity of brain networks to be robust or evolvable.
Flexibility	Propensity for brain nodes to dynamically switch their cluster affiliations.	Propensity for cognitive flexibility.
The brain is a network, like many other natural and synthetic systems.	The brain consists of connected elements, like many other natural and synthetic systems.	The brain shares functional network principles with many natural and synthetic systems.
Brain disorders are disconnection syndromes.	Brain disorders are correlated with brain-network abnormalities.	Brain disorders are caused by brain-network abnormalities.

First, *function* can denote physiological activity and also signal functional utility (Roux, 2014). The conflation of these two meanings may falsely attribute utility to all physiological phenomena. The heart pumps blood and makes lub-dub sounds, but only one of these actions is useful.

Second, *emergent* phenomena can denote higher-order structures in complex systems and also signal the importance of these structures (Bedau, 1997). The conflation of these two meanings may falsely attribute functional importance to higher-order structures. The structure of turbulent blood flow is emergent, but this flow plays no important role in heart function.

Third, *significance* can denote the rejection of a null hypothesis and also signal scientific importance (Wasserstein and Lazar, 2016). The conflation of these two meanings may falsely attribute importance to statistically significant features, especially if these features are also *functional* and *emergent*. In practice, the importance of a statistically significant result is strongly tied to the nature of the null hypothesis. A weak null hypothesis may propose, for example, that heart sounds are equally loud in still and beating hearts. We will always reject this null hypothesis, but such rejection will tell us little about the importance of heart sounds.

Collectively, the use of deepities can make speculative features seem useful or important. Moreover, the ability to fall back on the direct meanings of deepities in response to criticism, and to promote their implicit allusions at other times, can make deepities easy to defend and thus hard to eliminate. (This defense of deepities is known as “motte and bailey”, by analogy with a defense of a medieval castle [Shackel, 2005]. The motte is a hill with a tower — it is easily defensible but not particularly enjoyable to spend time in. The bailey is an outside court — it is enjoyable but not particularly defensible. The motte-and-bailey defense denotes a retreat to the motte in response to attacks and enjoyment of the bailey during more peaceful times.)

#### Suggestive structure: Spandrels

**Figure 5.**
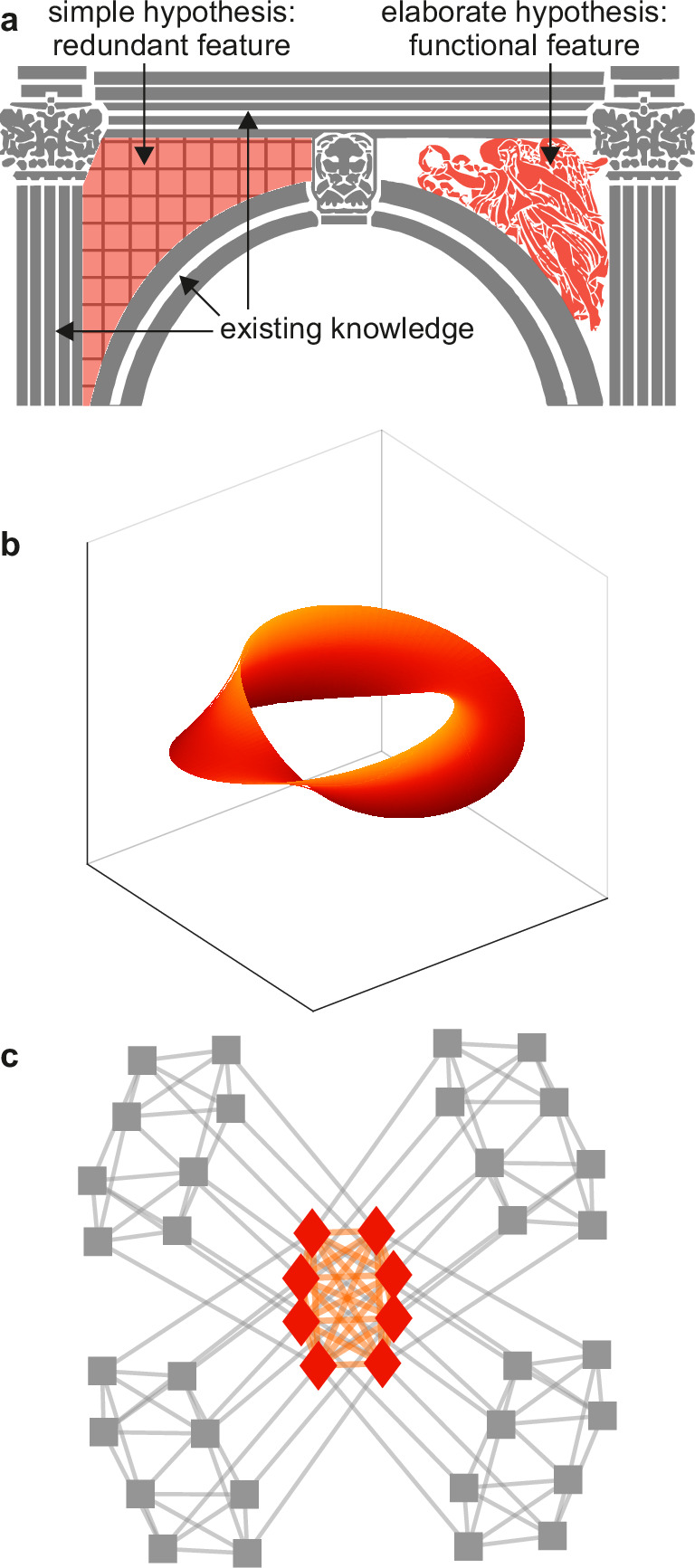
Example spandrels. (**a**) Spandrels in architecture denote triangular spaces of building arches (left, orange). Existing knowledge (gray) may explain these spaces as byproducts, but their intricate structure (right, orange) may suggest that they have important function. (**b**) An illustrative depiction of a “manifold” representation of neuronal population activity (orange). Axes denote directions of neuronal population activity in low-dimensional space. The intricate structure and predictive success of this feature may suggest that it plays an important role in neural function. The difficulty of testing this importance against existing knowledge (not shown) can make this importance speculative. (**c**) An illustrative depiction of a cortical core (orange). The intricate structure of this feature may suggest that it plays an important role in neural function. The relative ease of testing this importance against existing knowledge (gray) makes it possible to show that this feature is ultimately redundant. Panels (a) and (c) are adapted from (respectively) Figure 2b and Figure 1a of Rubinov, 2016.

In architecture, spandrels denote triangular spaces of building arches (Figure 5a). These spaces arise as byproducts of the contours of the arch, but their intricate decoration may suggest that they have important (decorative) function. In biology, spandrels are phenotypes that have intricate and similarly suggestive structure (Gould and Lewontin, 1979). For example, the intricate structure of turbulent lub-dub flow, and the ability of this flow to predict heart activity and physical exertion, may all suggest that lub-dub sounds play an important role in heart function. The intricate structure and predictive success of many features in systems neuroscience may likewise suggest that these features play an important role in brain function (Figure 5b–c).

The concept of spandrels helps to show the value of tests against benchmark models. For example, the lack of a benchmark model of vision makes it difficult to test the significance of internal representations against visuo-motor interactions (Figure 5b). This difficulty can make the existence and importance of internal representations inconclusive. Such inconclusiveness, in turn, may help to explain the vigorous and unsettled debates over the nature of this and other speculative features in systems neuroscience (Langdon et al., 2023; Sohal, 2016; Destexhe and Touboul, 2021). By contrast, well-defined benchmark models of cortical networks make it relatively easy to show the redundancy of cores or clubs against these models (Figure 5c). This ease may help explain the lack of comparable debates over the nature of these and other redundant features in network neuroscience (Liao et al., 2017; Sporns, 2018; Pasqualetti et al., 2019).

#### Suggestive narratives: Just-so stories

Just-so stories in biology are intriguing but speculative narratives that suggest the presence of theoretically elegant or optimal biological function (Gould, 1978; Bowers and Davis, 2012). A just-so-story may suggest, for example, that heart sounds exist to warn of overexertion and thus help minimize energy expenditure. Just-so stories can be difficult to falsify because it is often easy to reexplain some evident non-optimality as a globally optimal tradeoff between competing objectives (Gould and Lewontin, 1979). Table 3 shows examples of such stories in the recent systems-neuroscience literature.

**Table 3.** Example stories.

Concept	Initial narrative of optimality	Evidence of suboptimality (strong but unviable null model)	Restoration of optimality through the inclusion of an ad hoc tradeoff	Alternative benchmark narrative (strong and viable null model)
Criticality (Fontenele et al., 2019; Wilting and Priesemann, 2019; Nanda et al., 2023)	Brain activity always and exactly balances between order and disorder. This allows it to optimize information transmission and storage.	Brain activity does not always or exactly balance between order and disorder.	Brain activity optimizes the tradeoffs between the benefits of criticality and the competing benefits of flexibility or stability.	Brain activity avoids the extremes of overinhibition and overexcitation and is not optimal over and above this avoidance-of-extremes baseline.
Predictive coding (Sun and Firestone, 2020; Van de Cruys et al., 2020; Seth et al., 2020; Cao, 2020)	Brain activity aims to optimally predict incoming sensory input.	Brain activity optimally predicts sensory input in dark and quiet spaces. Despite this, animals tend not to seek out such spaces.	Brain activity aims to optimize the tradeoffs between predictions that are accurate and predictions that are motivational.	Brain activity reacts to sensory input but does not aim to optimally predict this input.
Wiring minimization (Markov et al., 2013; Bullmore and Sporns, 2012; Rubinov, 2016)	Brain-network structure globally minimizes wiring cost and therefore optimizes wiring economy.	Brain-network structure does not globally minimize wiring cost.	Brain-network structure optimizes the tradeoffs between wiring cost and communication efficiency.	Brain networks have long connections that enable specific sensory-motor function but do not optimize global communication.

The difficulty of falsifying just-so stories also helps to show the value of tests against benchmark models. Assertions of suboptimality form strong but unviable null models (Table 3, third column). Acceptance of these models, in other words, does not offer a viable alternative explanation to replace the original narrative. Without such a viable alternative, it becomes easy to hold on to the original narrative, typically by introducing an ad hoc tradeoff that restores optimality (Table 3, fourth column). This process may help to explain why just-so stories can hold sway in the field long after they are rejected against strong null models. By contrast, benchmark models form strong and viable null models (Table 3, fifth column). The acceptance of these models offers viable alternative explanations of brain function and, in this way, makes it easier to eliminate the original narrative (Appendix 2).

### Stagnation and progress

The commonness of circular analyses of knowledge can help explain a seeming disconnect between the fast pace of everyday discovery and the slow pace of real progress. Cobb, 2020 described the nature of this disconnect in neuroscience:

“There are now tens of thousands of brain researchers around the world, beavering away in a bewildering range of new subdisciplines […] each with their own questions, methods and approaches. Thousands of research articles relating to brain function appear each year.” Despite this, “[i]n reality, no major conceptual innovation has been made in our overall understanding of how the brain works for over half a century.”

On the one hand, circular analyses of knowledge can enable a fast pace of intriguing, and often replicable, everyday discoveries. On the other hand, the speculative and redundant nature of these discoveries does not lead to revisions of benchmark models and, in this way, results in a lack of real progress. Horgan, 2015 introduced the term “ironic science” to describe the nature of this process:

“Ironic science [acceptance of intriguing but speculative models] offers points of view, opinions, which are, at best, interesting, which provoke further comment. But it does not converge on the truth [lead to acceptance of truer models]. It cannot achieve empirically verifiable surprises that force scientists to make substantial revisions in their basic description of reality [make substantial revisions to benchmark models].”

Tests against benchmark models can help resolve this disconnect by ultimately linking the value of proposed discovery with revisions of benchmark models. Particle physics provides a good example of these tests in action. This field has the Standard Model, perhaps the most successful benchmark model in all of science today. The field seeks to revise this model but refreshingly accepts, and indeed embraces, the everyday failure to do so. Cousins, 2017 aptly summarized the nature of this practice:

“In many searches in [particle physics], there is a hope to reject the [Standard Model] and make a major discovery […]. But there is nonetheless high (or certainly non-negligible) prior belief in the null hypothesis. The literature, including the most prestigious journals, has many papers […] that report no significant evidence for the sought-for [beyond-the-Standard-Model] physics. Often these publications provide useful constraints on theoretical speculation, and offer guidance for future searches.”

In contrast to particle physics, benchmark models are often ill-defined in more expansive fields, such as psychology or sociology. The difficulty of evaluating real progress in these fields can make practitioners throw up their hands in despair (Yarkoni, 2020 gives an example from psychology). It may also make them avoid tests against null models altogether. For example, Gelman et al., 2020 noted:

“We do not generally use null hypothesis significance testing in our own work. In the fields in which we work [social science and public health], we do not generally think null hypotheses can be true [cf. strawman models can be truer than speculative models]. We do not find it particularly helpful to formulate and test null hypotheses that we know ahead of time cannot be true [cf. almost invariably accept speculative models against strawman models].”

Systems neuroscience probably lies somewhere between particle physics and social science. Some parts of the field, such as network neuroscience, are sufficiently circumscribed to allow tests of new models against well-delineated benchmark models. To be clear, it is unlikely that the field can converge on benchmark models that resemble the Standard Model or even remotely approach the explanatory success of this model. Despite these limitations, the adoption of routine tests against benchmark models can help place the field on a rigorous foundation and in this way facilitate real progress.

### Practical details

This section describes the practical details of testing new models against benchmark models. It first describes steps to integrate existing knowledge into benchmark models. It then discusses methods to sample data from benchmark-model distributions. It finally proposes practical steps to establish a culture of rigorous tests.

#### Integrating knowledge

Benchmark models should include all aspects of important existing knowledge about some phenomenon of interest. The need to include *all* knowledge reflects not dogma but the objective importance of control for *all* known confounding explanations. This need parallels the need to control for *all* aspects of the noise in tests on independent data (Appendix 3) or the need to control for *all* confounding explanations in randomized controlled trials (Box 2).

In principle, the inclusion of all important existing knowledge can seem daunting. In practice, however, this inclusion already happens routinely, albeit often informally, in books, reviews, and detailed Introduction sections of original articles. For example, an Introduction section that describes the importance of features *a*, *b,* and *c* to some phenomenon of interest, informally includes all these features in a benchmark model of this phenomenon.

Features that comprise important existing knowledge should rest on rigorous evidence from extensive observations or controlled experiments. Such evidence generally points to strong similarities between the nervous system and other body systems, and to strong similarities between the nervous systems of distantly related species. These similarities span functional objectives, structural building blocks, and developmental processes.

**Figure 6.**
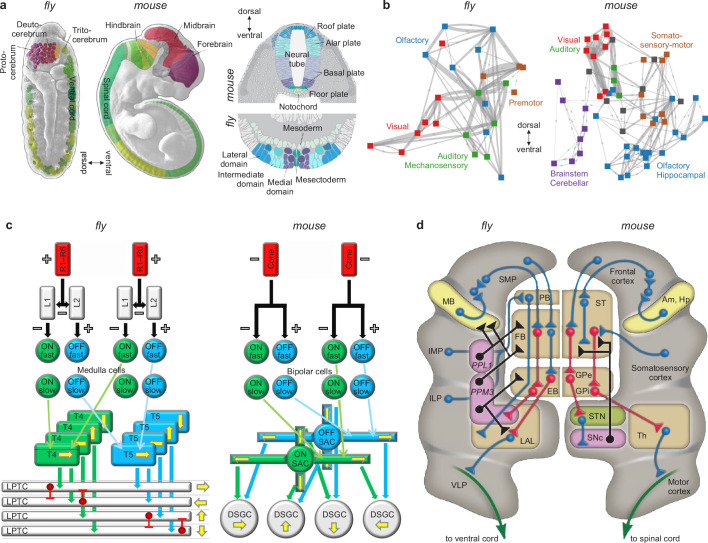
Similarities of development and structure in mice and flies. (**a**) Conserved rostrocaudal (nose-to-tail, left panels) and dorsoventral (back-to-belly, right panels) patterns of neural gene expression in developing flies and mice. Matching colors denote homologous genes. Gene names not shown. (**b**) Conserved gross organization of regional modules in adult flies and mice. Note that, relative to flies, the organization of (**a**) expressed neural genes and (**b**) visual, auditory, and olfactory modules in mice is inverted dorsoventrally. This is a known developmental quirk (Held, 2017). (**c**) Similarities in the motion-detection circuits of flies and mice. R1–R6 photoreceptors in flies, and cone photoreceptors in mice, convert light into neural activity. Each photoreceptor has a distinct receptive field that responds to spatially distinct light stimuli. Parallel ON and OFF pathways in both animals extract motion signals from this activity. These pathways start with L1/L2 lamina monopolar cells in flies, and directly with photoreceptors in mice. Cells in the ON pathway depolarize, and cells in the OFF pathway repolarize, in response to increased visual input. Moreover, distinct cells within each pathway may respond to input on fast or slow timescales. T4/T5 interneurons in flies, and starburst amacrine interneurons (SACs) in mice, detect motion in each pathway by integrating fast and slow responses associated with specific receptive fields. Finally, lobular plate tangential cells (LPTCs) in flies, and ON-OFF direction-selective ganglion cells (DSGCs) in mice, recombine motion signals from the ON and OFF pathways. +/− denote excitation/inhibition, and yellow arrows denote four directions of motion. (**d**) Proposed homologies between the action-selection circuits of flies and mice. The alignment emphasizes the shared function of individual areas and of excitatory or modulatory (blue), inhibitory (red), dopaminergic (black), and descending (green) projections. In flies, action selection centers on the central complex. The central complex includes the protocerebral bridge (PB), the fan-shaped body (FB), and the ellipsoid body (EB). In mice, action selection centers on the basal ganglia. The basal ganglia include the striatum (ST) and the external and internal globus pallidi (GPe and GPi). The central complex receives direct projections from sensory areas, the intermediate and inferior lateral protocerebra (IMP and ILP). It also receives direct projections from an association area, the superior medial protocerebrum (SMP). Finally, it receives indirect projections, via the SMP, from a learning area, the mushroom body (MB). Correspondingly, the basal ganglia receive direct projections from sensory and association areas in the cortex and indirect projections, via association cortex, from learning areas (the amygdala and hippocampus, Am and Hp). The central complex projects to the ventral cord via the lateral accessory lobes (LAL) and the motor ventrolateral protocerebra (VLP). Similarly, the basal ganglia project to the spinal cord via the thalamus and the motor cortex. Finally, in both cases, dopamine plays an important modulatory role. It acts via PPL1 and PPM3 neurons in flies, and via the substantia nigra pars compacta (SNc) in mice. Note also that the gall (not shown) may be a fly homolog of the mouse suprathalamic nucleus (STN, Fiore et al., 2015). Panel (a) is reproduced from Figure 1 of Bailly et al., 2013. Panel (b) is adapted from Figure 1b of Rubinov, 2016. Panel (c) is reproduced from Figure 5 of Borst and Helmstaedter, 2015. Panel (d) is adapted from Figure 2 of Strausfeld and Hirth, 2013.

We know, for example, that other body systems use effective but often inelegant tricks to solve diverse but always specific problems of survival and reproduction. We also know that nervous systems use similarly effective but inelegant tricks to feed, fight, flee, mate, and solve other diverse but similarly specific problems (Ramachandran, 1985; Marcus, 2009). We also know that the specific details of these tricks are similar in distantly related species (Nieuwenhuys and Puelles, 2016; Tosches, 2017; Cisek, 2019). These similarities include homologies of specific circuits (Sanes and Zipursky, 2010; Borst and Helmstaedter, 2015; Clark and Demb, 2016), systems (Strausfeld and Hirth, 2013; Fiore et al., 2015; Riebli and Reichert, 2016) and developmental processes (Carroll, 1995; Arthur, 2010; Held, 2017) in flies and mice, organisms that diverged about 600 million years ago (Figure 6). The importance, specificity, and conservation of these features make them natural candidates for inclusion in benchmark models (Appendix 5).

#### Defining models

*Models of the phenomena.* Benchmark models of relatively simple or circumscribed phenomena can sometimes take the form of parametric equations. In neuroscience, perhaps the best-known example of such a benchmark model is the Hodgkin-Huxley model of the action potential (Hodgkin and Huxley, 1952). By contrast, benchmark models of complex or expansive phenomena, such as whole-brain networks, are often hard to express in parametric form. These models can instead be defined pragmatically on the basis of benchmark features in empirical data (Table 4). Such data-driven definitions can resemble dimensionality reduction (Cunningham and Ghahramani, 2015) and force studies to formalize the often-vague theoretical concepts as quantifiable model features.

**Table 4.** Example features and statistics.

Model feature	Example statistic
Sensory-motor interactions	Connectivity and activity statistics of functional circuits.
Excitation/inhibition balance	1/*f* power-spectral slopes (Gao et al., 2017).
Node connectivity	Degree-distribution statistics (Clauset et al., 2009).
Network clusters	Within-module densities (Fortunato, 2010).
Tuning representations	Tuning-curve statistics (Kriegeskorte and Wei, 2021).
Manifold representations	Persistent-homology barcodes (Ghrist, 2008).
Oscillations	Frequency-specific amplitudes and phases (Donoghue et al., 2020).
Criticality	Avalanche exponents (Sethna et al., 2001).
Small worlds	Small-world statistics (Bassett and Bullmore, 2017).
Cores/clubs	Within-core densities (Csermely et al., 2013).
Network controllability	Network-controllability statistics (Pasqualetti et al., 2014).

Many applied or clinical fields seek to explain the nature of altered brain development, structure, or function. Formulation of benchmark models is equally important in these fields. Benchmark models of altered phenomena should correspondingly be defined in terms of altered, rather than absolute, values of empirical features. For example, benchmark models of neuropsychiatric disorders could be defined in terms of altered development and structure that coherently delineate specific patient populations (Insel and Cuthbert, 2015; Hampel et al., 2023).

*Models of the data.* In practice, benchmark models should also include features that represent data limitations or biases. For example, limitations of neural-activity data may include acquisition artifacts, physiological confounders and indirectness of neural-activity markers (Hillman, 2014; Wei et al., 2020). The inclusion of these data features in benchmark models can help to mitigate their confounding effects. The interactions of these features with other aspects of the signal, however, makes it ultimately impossible to fully eliminate these effects (Appendix 3).

#### Sampling data

Tests against benchmark models rest on the ability to sample data from benchmark-model distributions. This sampling should ideally be unbiased: the data samples should match the model statistics but be maximally random otherwise. Unbiased sampling allows us to make valid statistical inferences. For example, the opinions of an unbiased sample of people allow us to make valid statistical inferences about the opinions of the whole population.

In practice, fully unbiased sampling is often intractable, but approximately unbiased sampling is often possible for many interesting benchmark-model distributions. For clarity, this section distinguishes between specific and general methods for doing such sampling.

Specific sampling methods typically first express benchmark-model distributions as solution spaces of data that satisfy benchmark statistics (Schellenberger and Palsson, 2009). They then randomly draw data samples from these solution spaces. Important examples of these methods can sample data with spatial and temporal correlations (Prichard and Theiler, 1994; Roberts et al., 2016; Nanda et al., 2023). The main strength of these methods is in the ability to sample data in fast and unbiased ways. Their main weakness is the inability to sample data with general or arbitrary features and their consequent restriction to a relatively narrow set of benchmark models.

General sampling methods have a complementary set of strengths and weaknesses. The main strength of these methods is the ability to sample data with general or arbitrary benchmark features. Their main weakness is the slow or biased nature of the sampling.

General sampling methods comprise two broad types. The first type of general sampling typically begins with an initial data sample that typically matches the dimensionality and other basic properties of empirical data. It then iteratively randomizes this initial sample in a way that satisfies the benchmark statistics of empirical data, usually by minimizing an error function (Schreiber, 1998). Unbiased sampling requires that this randomization could, in principle, reach all possible samples and that randomization at each iteration could, in principle, be reversible (Newman and Barkema, 1999). These conditions imply that this randomization must be “non-greedy” or not necessarily lower the error at each iteration.

The second type of general sampling typically uses statistical inference methods, such as the principle of maximum entropy. It first defines and fits parametric data distributions and then randomly draws data samples from these distributions (Squartini and Garlaschelli, 2011). In contrast to other sampling methods, this approach preserves the benchmark statistics in the population average but not necessarily in each individual data sample. Fully unbiased sampling with this approach is often intractable for large datasets. Assumptions of independence can make this sampling tractable for many benchmark models but likely at the expense of considerable bias (Cimini et al., 2019).

#### Making progress

The importance of tests against benchmark models reflects the broader importance of scientific progress. In modern science, the notion of progress is intertwined with the concept of impact. Formally, impact often denotes the number of papers and citations. Implicitly, impact signals real progress. Circular analyses of knowledge enable speculative and redundant results that can lead to many intriguing, replicable, and highly cited papers. Such papers satisfy the formal meaning of impact even as they fail to make real progress (Lawrence, 2007; Alberts, 2013).

Tests against benchmark models can help to align the formal and intuitive definitions of impact. A narrow perspective on genuine impact could equate impact with direct revisions of benchmark models. A broader and more realistic perspective can also emphasize advances that indirectly facilitate revisions of benchmark models (Table 5).

**Table 5.** Examples of impactful advances.

Advance	Nature of impact
Discoveries	Revisions of benchmark models (typically rare).
Null results	Rejections of previously promising speculative models.
Exploratory advances	Formulations of newly promising speculative models.
Conceptual advances	Discoveries of explanatory gaps that enable exploratory advances.
Methodological advances	Improvements in data or analysis that support all the other advances.

Separately, the adoption of benchmarking best practices from predictive modeling fields, including machine learning (Weber et al., 2019; Mangul et al., 2019; Mitchell et al., 2019; Kapoor and Narayanan, 2023), can help facilitate progress in explanatory modeling. The following list describes three important examples of these practices:

1. High-quality and publicly accessible data can advance discovery in several ways. First, such data can serve as a reference for formulating consensus benchmark models. Second, such data can help reveal explanatory gaps in existing benchmark models. Third, such data can help formulate new and promising speculative models.
2. Standardized summaries of models and tests can help replace imprecise narratives with quantitative summaries of individual results. Machine-readable versions of these summaries can help facilitate automated integration of such results across studies.
3. A centralized integration of results can help to formalize discovery through continuous revisions of benchmark models. It can also help to collate and standardize null results and, in this way, eliminate rejected speculative models from future tests.

Together, this change in focus can help motivate systems neuroscientists to carefully formulate new models and to rigorously test these models against benchmark models. Such testing can lead to a welcome decrease in publications of speculative and redundant results. And collectively, the resulting alignment of formal and intuitive definitions of impact can give the field a better chance to make real progress.

### Concluding recommendations

Circular analyses of noise, and the resulting problem of irreplicable results, form a known impediment to progress in systems neuroscience. This work described that circular analyses of knowledge, and the resulting problem of redundant results, form a less-well known but similarly serious impediment. This concluding section summarizes my overall suggestions for resolving this problem. Appendix 6 discusses objections to some of these suggestions.

*Raise awareness.* Few scientists and funding bodies formally discuss the problem of redundant results. The lack of this discussion contrasts with extensive parallel discussions of the problem of irreplicable results. The start of this discussion, including in research and policy papers, will be an important first step towards the development of principled solutions.

*Reevaluate discoveries.* Systematic community efforts should establish the genuine novelty of discoveries in systems and network neuroscience. These efforts may benefit from the experience of similar efforts to establish the replicability of discoveries in psychological and social science (Open Science Collaboration, 2015; Camerer et al., 2018). These efforts face specific challenges, however, including establishing consensus on definitions of reference datasets, benchmark models, and test statistics.

*Delimit speculation.* Speculation often helps to formulate promising new models. At the same time, misuse of speculation can lead to the neglect of rigorous tests and to the inappropriate acceptance of speculative models. Studies should minimize this misuse by delimiting all suggestively speculative terms (deepities), structure (spandrels), and narratives (just-so stories). Ideally, these delimitations should be prominently made in Introduction sections.

*Define benchmarks.* Many parts of systems and network neuroscience lack benchmark models. The field should formulate such models to integrate all important existing knowledge and rigorously test proposed discovery. Challenges in the formulation of benchmark models include collation and curation of existing knowledge, consensus definition of model features and test statistics, and development of distinct models for individual phenomena.

*Advance sampling.* A dearth of powerful sampling methods limits the adoption of rigorous model tests. The field should develop unbiased and scalable methods for sampling data from diverse benchmark-model distributions. Challenges in the development of these methods include competing demands of unbiased sampling and scalability (for general methods) and extensions to diverse benchmark-model distributions (for specific methods).

*Reclaim impact.* The divergence of formal and intuitive meanings of impact can hinder scientific progress. A multifaceted assessment of direct or indirect impact that centers on revisions of benchmark models, and that discourages redundant explanations, can help to reduce this divergence. Research and funding bodies can emphasize this multifaceted assessment and downplay the use of publication metrics as indicators of progress.

## Figures and Tables

**Appendix 1—figure 1.**
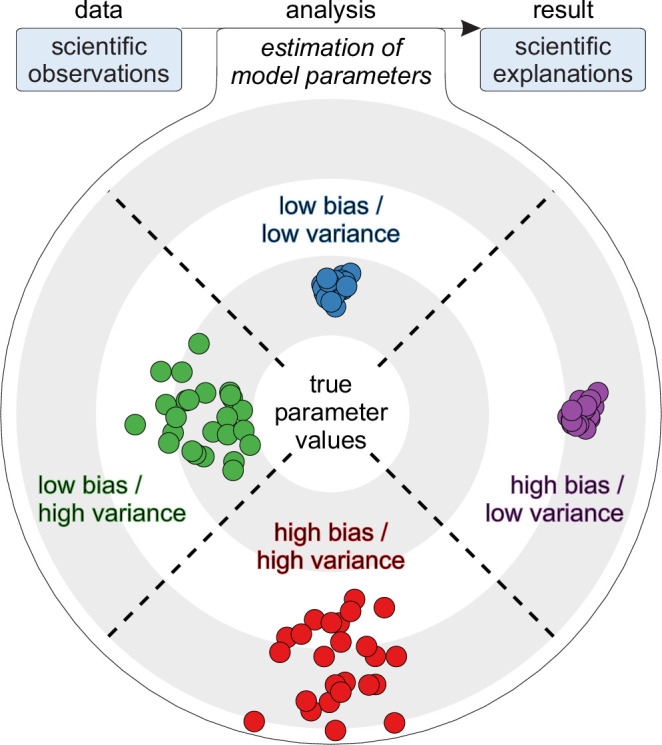
Statistical perspective on trueness and precision. Top (flowchart): Analyses as parameter estimates of explanatory models. Bottom (target): Four example parameter estimates with distinct precision and trueness profiles (colored dots). True parameter values denote true explanations and not true predictions. High-bias estimates denote explanations that have low relative trueness. By contrast, high-variance estimates denote explanations that have low precision.

**Appendix 5—figure 1.**
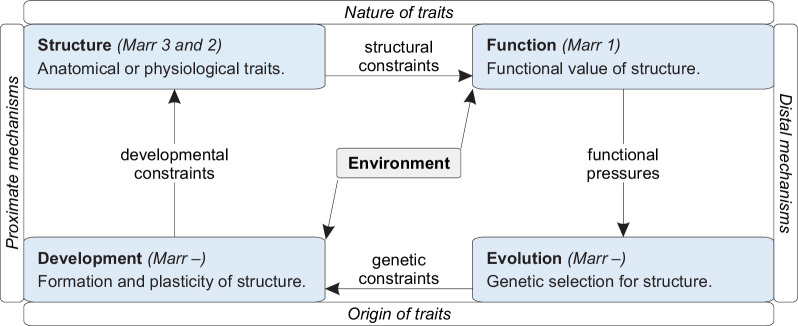
A framework for integrating existing knowledge. Tinbergen’s four levels of analysis (blue boxes) organized along dimensions that reflect Mayr’s distinction between proximal and distal mechanisms. Arrows denote interactions (pressures or constraints) between individual levels. Laland et al., 2011, Bateson and Laland, 2013, Krakauer et al., 2017, and Mobbs et al., 2018 provide additional discussions of this framework.

## Additional files

MDAR checklist

## Appendix 1

This section provides two complementary perspectives on the concept of relative trueness.

### Philosophical perspective

This work posits that all else being equal, models that are more explanatorily successful — that explain the data more accurately or with fewer assumptions — are likely to be truer than rival models. This position is largely compatible with the two main philosophical perspectives on model trueness (known in philosophy as truthlikeness [Oddie and Cevolani, 2022]). The most popular perspective, scientific realism, broadly posits that the most successful scientific models are likely to be approximately true (Bourget and Chalmers, 2014; Chakravartty, 2017). The main alternative perspective, scientific antirealism, broadly disagrees with this position. This disagreement forms the basis of a longstanding and possibly irreconcilable debate. Despite this disagreement, however, both perspectives broadly agree that all else being equal, more successful models are likely to be truer than rival models (Wray, 2010).

The position in this work is largely compatible with both perspectives because it narrowly centers on this point of agreement and because it avoids taking sides in the disagreement. Mizrahi, 2020 described a very similar middle-ground position of *relative realism*:

“[W]e have adequate grounds for believing that, from a set of competing scientific theories, the more empirically successful theory is *comparatively* true, that is, closer to the truth relative to its competitors in the set, rather than approximately true.” and “[A] scientific theory can be […] comparatively true, but still be quite far off from the truth.”

Relative trueness resembles the biological concept of relative fitness or reproductive success. Much as it is more meaningful to study the relative, rather than absolute, fitness of individual organisms (Orr, 2009), so it is often more meaningful to study the relative, rather than absolute, trueness of scientific models.

Note also that model trueness differs from model utility. For example, many models in neuroscience can make accurate predictions but be biologically unconstrained, artificially structured, or altogether uninterpretable. Such models are useful predictive tools (have high utility) but do not accurately explain biological reality (have relatively low trueness).

### Statistical perspective

This work focuses on scientific analyses that estimate parameters of explanatory models (Appendix 1—figure 1, top). Many such analyses cannot express explanatory models as parametric equations and cannot perform formal parameter estimation. Instead, these analyses often use significance tests to informally estimate parameters of underlying explanatory models.

This work assumes that all parameter estimates are directly comparable across all models. It makes this assumption without loss of generality because any two models can be nested within a more general model. It adopts the terminology of the International Organization for Standardization (ISO, 1994; Menditto et al., 2007) to quantify the accuracy of these parameter estimates. It defines trueness as the inverse of the estimation bias and precision as the inverse of the estimation variance (Appendix 1—figure 1, bottom). Note that trueness has a single and clear meaning, whereas bias is often a catch-all term, especially outside statistics (Danks and London, 2017; Fanelli et al., 2017).

This work focuses on the problem of redundant features in explanatory models. Successful explanatory models tend to have relatively low estimation bias. The inclusion of redundant features tends to increase this bias and thereby reduce explanatory success. By contrast, the work does not consider the problem of redundant features in predictive models. Successful predictive models have relatively low prediction bias but may not necessarily have low estimation bias, as we saw in the above distinction between trueness and utility. The inclusion of redundant features in these models does not necessarily increase their prediction bias (Guyon and Elisseeff, 2003) and, in this way, does not necessarily reduce their predictive success. (Note that this distinction between explanatory and predictive modeling differs from the treatment of Shmueli, 2010).

## Appendix 2

This section relates circular and unified analyses to Mayo’s framework of severe testing (Mayo and Spanos, 2011; Mayo, 2018). First, it shows that circular analyses of knowledge form a specific violation of Mayo’s weak-severity requirement. Second, it shows that unified analyses form a specific adherence to Mayo’s strong-severity requirement. Third, it describes unified analyses as a type of *severe selection*: a hybrid approach that combines severe testing with model selection.

### Weak-severity requirement and circular analysis

Mayo, 2018 defines her weak-severity requirement as follows:

“One does not have evidence for a claim if nothing has been done to rule out ways the claim may be false. If data […] agree with a claim C but the method used is practically guaranteed to find such agreement, and had little or no capability of finding flaws with C even if they exist, then we have bad evidence, no test (BENT).”

Appendix 2—table 1 contrasts this definition with our definitions of circular analysis.

**Appendix 2—table 1.** Weak-severity requirement and circular analysis.

Weak-severity requirement (Mayo)	Circular analysis (this work)
*Bad evidence, no test*. Use a test that practically guarantees to find agreement between data and claim and has little or no capability of finding flaws with the claim even if they exist. Show that data agree with the claim on the basis of this test.	*General definition (weak evidence of progress*). Test a model in a way that almost invariably accepts the model. Accept the model on the basis of this test.
*N/A*. The framework of severe testing, and the weak-severity requirement, do not specifically consider the problem of redundant explanations.	*Specific definition (strong evidence of stagnation*). Test the statistical significance of redundant features in a way that almost invariably shows this significance against a strawman model. Accept the corresponding model on the basis of this test.

### Strong-severity requirement and unified analysis

Mayo, 2018 defines her strong-severity requirement as follows:

“We have evidence for a claim C just to the extent it survives a stringent scrutiny. If C passes a test that was highly capable of finding flaws or discrepancies from C, and yet none or few are found, then the passing result […] is evidence for C.”

Appendix 2—table 2 contrasts this definition with our discussion of unified analysis.

**Appendix 2—table 2.** Strong severity and unified analysis.

Strong-severity requirement (Mayo)	Unified analysis (this work)
*Evidence from survival of stringent scrutiny*. Use a test that is highly capable of finding flaws or discrepancies with a claim if they exist. Show that this test does not find flaws or discrepancies with the claim.	*Evidence of genuinely new discovery*. Define a benchmark model that includes all important existing knowledge about some phenomenon of interest. Show the statistical significance of a speculative feature against this model.

### Severe selection

Severe testing largely builds on Popper’s ideas and terminology. Popper, 1963 considered that falsification centers on genuine or severe tests:

“A theory which is not refutable by any conceivable event is nonscientific. Irrefutability is not a virtue of a theory (as people often think) but a vice. Every genuine [or severe] test of a theory is an attempt to falsify it, or to refute it. Testability is falsifiability.”

At the same time, Popper did not consider that severe tests need to involve testable, or viable, rival models, “the negation of a testable (or falsifiable) statement need not be testable” (Popper, 1963). For example, a wrong prediction can falsify a speculative model but need not accept a testable or viable rival model.

This lack of viable rival models can make it hard to eliminate falsified explanations (Table 3). Lakatos, 1976, among others (Musgrave, 1973), has made this point:

“‘Falsification’ […] (corroborated counterevidence) is not a sufficient condition for eliminating a specific theory: in spite of hundreds of known anomalies we do not regard [a theory] as falsified (that is, eliminated) until we have a better one.”

By contrast, tests against benchmark models always lead to the acceptance of viable rival models. In this sense, these tests form a type of severe selection: they combine aspects of severe testing (stringent scrutiny) with aspects of model selection (viable rival models). This severe selection between strong rival models may also resemble, more closely than severe testing, the natural selection of strong rival organisms (Appendix 1).

## Appendix 3

This section provides a unified perspective on the circular analyses of noise in Kriegeskorte et al., 2009 and the circular analyses of knowledge in this work. It shows that these two problems, and their corresponding solutions, share deep similarities but also have basic differences. The differences reflect the basic distinctions between overfitting and overspecification.

### Similarities of circular analyses

Kriegeskorte et al. described circular analyses that lead to overfitting — the corruption of results by noise. This work describes circular analyses that lead to overspecification — the confounding of results by existing knowledge. We can show that these two problems are equivalent in important respects, by translating parts of Kriegeskorte et al. (slightly edited for clarity) into the language of this work. For simplicity, this translation focuses on an extreme version of overfitting, the full redundancy (rather than the mere non-independence) of model features with noise. Underlined text in this translation highlights the differences with the main text.

As in the main text, we can formally define this problem with three types of models.

*Benchmark models.* These models represent all important assumptions about noise in the data. In most cases, we may simply assume that the data have noise. In some other cases, we may also assume that noise in the data follows a specific distribution.

*Kriegeskorte et al.*: “Data are always a composite of true effects and noise.”
*Translation*: The benchmark model assumes that the data have noise.

*Speculative models.* These models include one or more speculative features of possible but uncertain importance. Some of these speculative features may be redundant with benchmark (noise) features. In practice, these redundant features will strongly correlate with noise in the data.

*Kriegeskorte et al.*: “A model may capture the noise to some extent as its parameters are fitted to the data.”
*Translation*: A model may include speculative features that are redundant with benchmark (noise) features.

*Strawman models*. These models represent weak null hypotheses. Kriegeskorte et al. do not consider strawman models in their discussion. Here, we can equate the absence of strawman models with the presence of maximally weak strawman models.

These definitions allow us to express the problem in Kriegeskorte et al. in our language.

*Circular analyses and irreplicable explanations (overfitted models*). Circular analyses almost invariably show the significance of speculative features against a strawman model because:

The speculative feature is redundant with one or more benchmark (noise) features.
The maximally weak (absent) strawman model excludes the benchmark (noise) features with which the speculative features are redundant.

These analyses implicitly accept a new benchmark model that includes all existing benchmark (noise) features as well as the new redundant speculative features. In this way, these analyses explain the same aspect of the data twice: first as assumptions about noise and second as a new discovery redundant with these assumptions.

*Kriegeskorte et al.* (Supplementary Discussion): “Using the same data set to generate and test a hypothesis is circular unless [we] address the question: If the data contained only noise and we searched for an effect the way we did, with what probability would we find an effect as strong as (or stronger than) the one we observed?”
*Translation*: Accepting a feature known to be redundant with noise is circular unless we test the significance of the feature against a benchmark (noise) model.

### Similarities of unified analyses

Kriegeskorte et al. described two tests to counter circular analyses of noise. Both tests center on the sampling of data and on testing the significance of speculative features against these sampled data. We can likewise translate these descriptions (slightly edited for clarity) into the language of this work.

*Tests of non-redundancy.* These tests are equivalent to our tests against benchmark models. First, they sample data from benchmark-model distributions: data in which all benchmark (noise) features are preserved, and all other aspects of the data are maximally random. Second, they test the statistical significance of speculative features against these data. Third, the finding of statistical significance implies that the tested speculative features are not redundant with benchmark (noise) features.

*Kriegeskorte et al.* (Supplementary Discussion): “Modeling the effect of assumptions may not be tractable analytically, but could be achieved by simulation of null data.”
*Translation*: Testing the non-redundancy of speculative features against benchmark (noise) features may not be tractable analytically, but could be achieved by sampling data from benchmark (noise) model distributions.

*Tests of independence.* These tests are not discussed in the main text. First, they sample data in which all benchmark (noise) features are maximally random, and all other aspects of the data are preserved. Second, they test the statistical significance of speculative features against these data. Third, the finding of statistical significance implies that the tested speculative features are independent of benchmark (noise) features.

*Kriegeskorte et al.* (Supplementary Discussion): “Independent data can ensure independence of the results under the null hypothesis.”
*Translation*: Data in which all benchmark (noise) features are maximally random (independent), and all other aspects of the data are preserved can ensure the independence of speculative features from benchmark (noise) features.

*Conceptual considerations.* Kriegeskorte et al. primarily advocate tests of independence to prevent circular analyses of noise. Our discussion helps us to appreciate the reason for this advocacy. Noise is, by definition, an unwanted feature of the data. Therefore, it is important to show that a speculative feature is independent of noise rather than merely not redundant with it. Tests of independence, but not tests of non-redundancy, can allow us to achieve this goal.

*Kriegeskorte et al.* (Supplementary Discussion): “Tests on null data from a random generator [of noise] can help catch statistical circularities. Unfortunately, the absence of a bias in such tests does not indicate that analyses are noncircular.”
*Translation*: Tests of non-redundancy can help catch statistical circularities by showing that the speculative features are redundant with the noise features. Unfortunately, the absence of redundancy in such tests does not indicate that the speculative features are independent of the noise features.

*Practical considerations.* The irreplicable nature of noise and the replicable nature of existing knowledge have practical implications for tests of independence. Specifically, the acquisition of new data under the same experimental conditions simulates the sampling of data in which all benchmark (noise) features are maximally random (independent), and all other aspects of the data are preserved. It follows that such data can be used to test the independence of speculative features from noise but not the independence of these features from existing knowledge.

*Kriegeskorte et al.* (Supplementary Discussion): “Independence in this context means the noise is statistically independent between the two data sets but real effects in the data will replicate.”
*Translation*: Independent data amounts to the sampling of data in which all benchmark (noise) features are maximally random, and all other aspects of the data are preserved.

### Summary of similarities

Circular analyses of noise and circular analyses of knowledge have the same basic structure. First, these analyses are vulnerable to distortions by extraneous features. Second, these analyses neglect to test for these distortions. Third, and due to this neglect, both analyses almost invariably explain the same aspect of the data twice.

Appendix 3—table 1 summarizes these basic similarities (underlined text highlights the main differences).

**Appendix 3—table 1.** Two types of circular analysis.

	Circular analysis of noise	Circular analysis of knowledge
Conceptual problem	Explanation of the same aspect of the data twice: first as noise, and second as a new discovery non-independent of this noise.	Explanation of the same aspect of the data twice: first as existing knowledge, and second as new discovery redundant with this knowledge.
Statistical problem	Model overfitting that results in high variance (low precision) of estimated model parameters.	Model overspecification that results in high bias (low trueness) of estimated model parameters.
Statistical solution	Tests of independence against sampled data in which all benchmark (noise) features are maximally random and all other aspects of the data are preserved.	Tests of non-redundancy against sampled data in which all benchmark (existing knowledge) features are preserved and all other aspects of the data are maximally random.

*Use novelty, theoretical novelty, and double dipping.* Kriegeskorte et al. described circular analyses as a form of double dipping — the use of the same aspect of the data to formulate and test new models. This process violates the requirement for (data) use novelty (Worrall, 1978). Our discussion described a more general problem of double dipping: the explanation of the same aspect of the data twice — as a benchmark feature and as a redundant feature. This more general problem violates the requirement for theoretical novelty — the need to transcend existing explanations of the data (Mayo, 2018).

### Analyses of artifact

We can consider data artifact as another extraneous feature. Artifact has distinct properties to noise and to existing knowledge. On the one hand, artifact is like noise because it is an unwanted feature of the data. On the other hand, artifact is like existing knowledge because it can replicate under the same experimental conditions. Together, these properties suggest that neither of the above tests can fully show the independence of results from artifact. In practice, and to mitigate this problem, we can try to remove artifact from data or test results on data recorded under different experimental conditions (Geirhos et al., 2020).

### Terminology

Finally, note that assumptions about artifact or noise also form a type of existing knowledge. In this sense, a more accurate, but somewhat more cumbersome, terminology of circular analyses could distinguish between:

- Circular analyses of [existing knowledge of] artifact.
- Circular analyses of [existing knowledge of] noise (the focus of Kriegeskorte et al.).
- Circular analyses of [existing knowledge of] signal (the focus of this work).

## Appendix 4

### Literature selection

I first searched the Web of Science database in January 2019 for all original articles that contained the following topic terms (TS):

(TS=("network neuroscience"))
 OR 
((TS=("connectom*"))
 AND 
(TS=("analy*") OR TS=("model*")))
 OR 
((TS=("*brain*") OR TS=("*cort*"))
 AND 
(TS=("network theor*") OR TS=("network analy*") OR TS=("network topolog*") OR TS=("network control*") OR TS=("graph theor*") OR TS=("complex network*")))

The initial search produced thousands of articles. I restricted this search to all articles that were published between 2014 and 2018 in neuroscience (*Nature Neuroscience*, *Neuron*), life science (*eLife*, *PLOS Biology*), clinical (*Brain*, *Biological Psychiatry*), or multidisciplinary (*Nature*, *Science*, *Nature Communications*, *PNAS*) journals. This restricted search produced 235 articles.

### Literature evaluation

I manually evaluated the Methods and Results sections of all structured articles, or the full text of all unstructured articles, for the presence of circular analyses of knowledge. This evaluation centered on the following three conditions.

- Condition 1: Presence of at least one network-neuroscience model. Network neuroscience models are network-science models of brain networks. By convention, I excluded standard dimension-reduction models of networks, such as principal component analysis, and standard network-inference models, such as dynamic causal models.
- Condition 2: Acceptance of at least one *M*_1_, where:
  - *M*_1_ is a network-neuroscience model of the studied data.
  - *M*_1_ includes a feature *X*_1_ that represents some function *F*_1_.
  - There is no strong known mechanistic link between *X*_1_ and *F*_1_.

- Condition 3: No test of *M*_1_ against at least one *M*_0_, where such a test is possible, and where:
  - *M*_0_ is a model of the same studied data.
  - *M*_0_ includes only features with known mechanistic links to function.
  - *M*_0_ is known or likely to explain *X*_1_ as a redundant feature.

### Results

This analysis found that 61% of all evaluated studies had at least one network-neuroscience model (satisfied Condition 1). These studies were suitable for further evaluation. Of these studies, 56% had at least one circular analysis of knowledge (satisfied Conditions 2–3). This estimate has a 95% bootstrap uncertainty interval of [48%, 64%]. Another 10% of studies may or may not have had such analyses. I could not say with certainty if these additional studies accepted an *M*_1_ or tested it against a possible *M*_0_.

I revisited this search in January 2023 to include all journal articles that contained the same topic terms and that were published between 2013 and 2022. This additional search yielded 11,395 articles. I extrapolated the above percentages to estimate that more than three thousand (11 thousand × 0.61 × 0.56) studies in this larger set had at least one circular analysis of knowledge.

### Auxiliary pipeline

To facilitate future systematic assessment of circular analyses of knowledge, I created an extensible semi-automated analysis pipeline in Python, a popular programming language. The following text summarizes the individual steps in this pipeline:

- Step 1: Environment set-up and loading of previously analyzed data.
- Step 2: Specification of the full literature-search query and instructions for manually downloading all reference records that match this query from the *Web of Science*.
- Step 3: Automated download of all full-text articles that match the specified search query.
- Step 4: Automated curation and cleaning of study text for all downloaded articles.
- Step 5: Automated extraction of relevant text segments and emphasis of potential keywords.
- Step 6: Automated scoring of the presence or absence of circular analyses based on manual evaluation of specified criteria.
- Step 7: Automated storage of collated evaluations and scores in a simple database and a summary table.

### Resource availability

Data and code are available at https://github.com/mikarubi/litrev (copy archived at Rubinov, 2023).

## Appendix 5

This section describes a framework for integrating existing knowledge about the function, structure, development, and evolution of individual biological features or traits (Appendix 5—figure 1). A benchmark model of a specific phenomenon should include, where possible, important existing knowledge from all aspects of this framework.

This framework is based on classifications of Mayr and Tinbergen (Mayr, 1961; Tinbergen, 1963; Laland et al., 2011; Bateson and Laland, 2013; Nesse, 2013) and is organized along two dimensions. The first dimension reflects the nature and origin of a trait. It distinguishes what the trait is (structure and function) from how it came to be (development and evolution). The second dimension reflects the mechanistic timescales of this nature and origin. It distinguishes the proximate mechanisms of a single lifetime (structure and development) from the distal mechanisms of many generations (function and evolution).

This framework is widely accepted in biology but less well-known in neuroscience. Instead, more neuroscientists seem to know about Marr’s (and Poggio’s) three-level framework for studying the brain as a computer (Marr, 1982). The first level of this framework (Marr 1) denotes the aim of brain “computation”. The second level (Marr 2) denotes the “algorithms” that achieve this aim. The third level (Marr 3) denotes the “hardware” that implements the algorithms.

The focus on computation alone is somewhat restrictive because it separates the brain from the rest of the body. We have no rigorous evidence to support this separation (see the main text for more discussion). Interestingly, and in line with this observation, Poggio recently updated Marr’s framework to include development and evolution (Poggio, 2012). In this way, he seems to have independently converged on Tinbergen’s more general biological classification.

## Appendix 6

This section discusses possible objections to the main recommendations in this work.

### Benchmark models are complicated

*Objection.* Benchmark models that include all important existing knowledge will often be complicated. The acceptance of such models violates the scientific preference for simplicity.

*Clarification.* There is an important difference between the preference for simplicity and the preference for parsimony. The preference for simplicity asserts that successful scientific models should be simple or elegant. This preference can be appealing but is not objectively defensible. Simple or elegant models can be aesthetically pleasing and can help formulate speculative models, but we have no objective evidence that they provide the most successful explanations of reality. van Fraassen, 1980 made this point more forcefully:

“[S]ome writings […] suggest that simple theories are more likely to be true. But it is surely absurd to think that the world is more likely to be simple than complicated (unless one has certain metaphysical or theological views not usually accepted as legitimate factors in scientific inference).”

By contrast, the preference for parsimony asserts that all else being equal, models with fewer redundant features are likely to be more explanatory successful (or truer) than rival models. This principle makes no assumptions about simplicity or elegance and, in this way, merely embodies aspects of rational thinking.

The distinction between simplicity and parsimony has real implications for scientific practice. For example, the Standard Model of particle physics is parsimonious insofar as it lacks redundant features. Despite this, this model is neither simple nor elegant — instead, particle physicists have called it “ugly”, “repulsive”, and “awkward” (Oerter, 2006). On this basis, proponents of simplicity should eliminate the Standard Model from scientific practice. By contrast, proponents of parsimony can accept this model as a successful benchmark.

Simplicity and parsimony may coexist in benchmark models of simple or circumscribed phenomena. The Hodgkin-Huxley model is both relatively simple and parsimonious. By contrast, simplicity and parsimony are less likely to coexist in benchmark models of complex or expansive phenomena, including in models of whole-brain networks.

### Benchmark models favor reductionist explanations

*Objection.* Benchmark models ignore that the same biological structure can have many functions. Specifically, these models tend to favor reductionist features and prevent the acceptance of emergent features. In practice, however, reductionist features can coexist with or give rise to important emergent features. For example, sensory-motor circuits can coexist with or give rise to important internal representations.

*Clarification.* Benchmark models can attribute multiple functions to the same biological structure as long as these attributions produce non-redundant explanations of the data. The showing of such non-redundancy, in turn, requires rigorous evidence. We often have such evidence for reductionist features. By contrast, we often lack such evidence for emergent features, because emergent features are often hard to test in controlled ways.

A more general objection of this sort may appeal to intuitions. It may assert, for example, that emergent brain function is intuitively distinct from reductionist body function or that emergent human cognition is intuitively distinct from reductionist insect cognition. All such intuitions likewise require rigorous evidence. This evidence can be experimental or computational. It cannot, however, be solely speculative.

### Benchmark models are ill-defined

*Objection.* Benchmark models are ill-defined for many expansive phenomena. In basic neuroscience, for example, a benchmark model of cognition is ill-defined because cognition has a wealth of distinct meanings. Similarly, in clinical neuroscience, a benchmark model of schizophrenia is ill-defined because this disorder has a wealth of heterogeneous pathology.

*Clarification.* The ill-defined nature of benchmark models reflects the ill-defined nature of many expansive phenomena. Improved definitions of these phenomena can naturally lead to improved definitions of the corresponding benchmark models. One approach to improve these definitions could focus on narrower and better-delineated portions of individual expansive phenomena. This approach could, for example, focus on well-delineated cognition linked to specific sensory-motor function or on well-delineated developmental changes linked to symptoms of schizophrenia. Iterative revisions of these narrower definitions can ultimately converge on well-defined phenomena and well-defined benchmark models.

### Descriptive, explanatory, generative and null models are distinct

*Objection.* On the one hand, many speculative models are descriptive and not explanatory models and therefore need not be rigorously tested. On the other hand, benchmark models are generative and not null models and therefore should not be used to test other models.

*Clarification.* The nature of outwardly different model types is often similar or equivalent. The assignment of distinct roles to these equivalent models is redundant in much the same way as the assignment of distinct function to equivalent features.

First, descriptive models in neuroscience should, in many cases, be more properly termed explanatory models. Strictly speaking, descriptive models should provide neutral summaries of data. In neuroscience, however, “descriptive” summaries often represent hypotheses about important aspects of brain structure or function. In these cases, descriptive models essentially do the work of explanatory models.

Second, explanatory models can typically generate data with few or no additional assumptions. For example, an explanatory benchmark model specifies a distribution of data samples that match the empirical benchmark statistics. The sampling of data from these distributions generates data.

Third, generated data of explanatory benchmark models can be used to test null hypotheses. This finally underscores the equivalence of explanatory, generative, and null models.

In practice, “generative models” in the literature often have features that allow the sampling of data relatively easily (without need for computationally intensive methods). In some cases, these features may reflect existing knowledge. In other cases, they do not reflect existing knowledge and therefore reflect sampling bias. Separately, “null models” in the literature often lack existing knowledge and, as a consequence, are easy to reject. The exclusion of existing knowledge from these models underpins the problem of circular analyses of knowledge.

### Tests against benchmark models favor old knowledge

*Objection.* Tests against benchmark models prevent interesting new discoveries. In an extreme case, a parsimonious benchmark model that perfectly explains some phenomenon of interest will be very hard to reject. The inability to reject this model will stifle progress.

*Clarification.* Tests against benchmark models help to prevent false new discoveries. This is not a weakness but a strength of these tests. Greater and greater knowledge can make it harder and harder to make new discoveries (because a previously made discovery cannot be new again). The difficulty of rejecting stronger and stronger benchmark models merely formalizes this process. The inability to reject benchmark models can still be impactful, however, as null results that may facilitate future discoveries (see the main text for additional discussion).

### Tests against benchmark models are hard

*Objection.* Tests against benchmark models require the sampling of data from complex benchmark-model distributions. Such sampling is often slow and sometimes intractable.

*Clarification.* The slowness of data sampling needs to be placed in context. Many current tests against strawman models take negligible time, especially relative to data acquisition or analysis. The commonness of speculative and redundant explanations suggests that this negligible allocation of time is unjustified. Tests against benchmark models, like other controlled experiments (Box 2), provide rigorous evidence of new discovery. The slowness of such tests is generally compensated by the strength of this evidence.

In some cases, sampling from benchmark-model distributions may be simply intractable. In such cases, it may still be possible to rigorously test speculative models in other ways. For example:

1. We could test if speculative models can make specific and surprising predictions. This approach may allow us to severely test individual models but does not necessarily offer viable alternative models or test explanatory model success (Table 3, Appendix 1–2). In addition, the ability to devise surprising and testable predictions may be nontrivial for complex neuroscience phenomena.
2. We could estimate maximum likelihoods and quantify the trade-offs between complexity (number of parameters) and agreement with data (likelihood) of competing models (Aho et al., 2014). This approach may allow us to select between models but does not necessarily offer severe tests if all the competing models are strawmen (Appendix 2). In addition, the estimation of maximum likelihoods may often be nontrivial, especially for models of large datasets.
3. We could bypass numerical tests by showing analytical equivalences of outwardly distinct model features. The approximate equivalence between node connectivity and average controllability is one example of such an analytical equivalence. Analytical equivalences can be elegant and instructive, but their discovery is generally idiosyncratic and is often intractable, especially for complex or highly nonlinear models.

In many cases, we lack the ability to rigorously test interesting models. In these cases, we need to acknowledge that such untestable models — no matter how elegant, intuitive, or appealing — ultimately remain speculative.
